# Unconventional excitonic states with phonon sidebands in layered silicon diphosphide

**DOI:** 10.1038/s41563-022-01285-3

**Published:** 2022-06-16

**Authors:** Ling Zhou, Junwei Huang, Lukas Windgaetter, Chin Shen Ong, Xiaoxu Zhao, Caorong Zhang, Ming Tang, Zeya Li, Caiyu Qiu, Simone Latini, Yangfan Lu, Di Wu, Huiyang Gou, Andrew T. S. Wee, Hideo Hosono, Steven G. Louie, Peizhe Tang, Angel Rubio, Hongtao Yuan

**Affiliations:** 1 National Laboratory of Solid State Microstructures, Jiangsu Key Laboratory of Artificial Functional Materials, College of Engineering and Applied Sciences, and Collaborative Innovation Center of Advanced Microstructures, Nanjing University, Nanjing, China; 2 Max Planck Institute for the Structure and Dynamics of Matter, Center for Free Electron Laser Science, Hamburg, Germany; 3 Department of Physics, University of California, Berkeley, CA USA; 4 Materials Sciences Division, Lawrence Berkeley National Laboratory, Berkeley, CA USA; 5 School of Materials Science and Engineering, Peking University, Beijing, China; 6 Materials Research Center for Element Strategy, Tokyo Institute of Technology, Yokohama, Japan; 7 College of Materials Science and Engineering, National Engineering Research Center for Magnesium Alloys, Chongqing University, Chongqing, China; 8 Center for High Pressure Science and Technology Advanced Research, Beijing, China; 9 Department of Physics, National University of Singapore, Singapore, Singapore; 10 School of Materials Science and Engineering, Beihang University, Beijing, China; 11 Center for Computational Quantum Physics, Simons Foundation, Flatiron Institute, New York, NY USA

**Keywords:** Two-dimensional materials, Electronic properties and materials, Electronic structure, Optical spectroscopy

## Abstract

Complex correlated states emerging from many-body interactions between quasiparticles (electrons, excitons and phonons) are at the core of condensed matter physics and material science. In low-dimensional materials, quantum confinement affects the electronic, and subsequently, optical properties for these correlated states. Here, by combining photoluminescence, optical reflection measurements and ab initio theoretical calculations, we demonstrate an unconventional excitonic state and its bound phonon sideband in layered silicon diphosphide (SiP_2_), where the bound electron–hole pair is composed of electrons confined within one-dimensional phosphorus–phosphorus chains and holes extended in two-dimensional SiP_2_ layers. The excitonic state and emergent phonon sideband show linear dichroism and large energy redshifts with increasing temperature. Our ab initio many-body calculations confirm that the observed phonon sideband results from the correlated interaction between excitons and optical phonons. With these results, we propose layered SiP_2_ as a platform for the study of excitonic physics and many-particle effects.

## Abstract (web summary)

Distinct electronic and optical properties emerge from quantum confinement in low-dimensional materials. Here, combining optical characterization and ab initio calculations, the authors report an unconventional excitonic state and bound phonon sideband in layered silicon diphosphide.

## Main

An exciton, the electron–hole pair formed via Coulomb interaction, is an ideal platform for understanding many-body effects^1–8^. The properties of excitons strongly depend on the crystal structure and dimensionality of host materials^9,10^. Due to quantum confinement, the electronic properties of quasiparticles (electrons, holes and excitons) in low-dimensional materials can be remarkably different from those in three-dimensional (3D) bulk materials. The Coulomb screening in low-dimensional quantum-confined structures, particularly in one-dimensional (1D) electronic systems, is known to be weaker than that in bulk systems and consequently leads to larger exciton binding energy^11–14^ and other emergent excitonic phenomena^10^. Experimental observations of anisotropic excitons have been demonstrated in two-dimensional (2D) van der Waals (vdWs) materials in which electrons and holes taking part in the formation of 2D excitons are confined in the same monolayer^15–19^. Meanwhile, in 1D materials such as carbon nanotubes (CNTs)^12,20^, 1D excitonic states have also been observed in which constituent electrons and holes are known to be confined within 1D nanostructures. A unique excitonic state with hybrid dimensionality, which is yet elusive, such as a bound electron–hole pair with an electron confined along one dimension (1D-confined electron) and a hole confined along two dimensions (2D-confined hole), or vice versa, would be of great interest and highly desired in terms of its optical properties and interactions with other emergent quasiparticles.

In this work, we demonstrate the observation of an unconventional bright exciton in a layered silicon diphosphide (SiP_2_) crystal, accompanied by a correlated phonon sideband in the optical spectrum. Based on our ab initio many-body *GW* and *GW* plus Bethe–Salpeter equation (*GW*–BSE) calculations, as well as non-perturbative model calculations, we find that the electrons constituting the excitons are confined within the 1D phosphorus–phosphorus chains of SiP_2,_ while the correlated holes extend over the 2D SiP_2_ atomic plane. Therefore, excitonic states in layered SiP_2_ are expected to exhibit hybrid dimensionality properties. Photoluminescence (PL) spectroscopy and reflectance contrast (RC) spectroscopy show that, regardless of the polarization of the excitation laser, the optical response of the excitonic state is always linearly polarized along the *x* direction of the SiP_2_ lattice and is accompanied by a unique sideband feature. Both the excitonic emission and the sideband feature undergo dramatic redshifts as the temperature increases, in contrast to a slight temperature-dependent redshift of the band edge that is mainly influenced by electron–phonon coupling^21,22^. This reveals that in SiP_2_ the interaction of the electronic degrees of freedom with the phononic degrees of freedom is strongly enhanced by excitonic effects. The phonon sideband feature can be theoretically modelled using a non-perturbative approach to describe the interaction between the unconventional excitons and optical phonon modes. Note that reduced dimensionality normally leads to excitonic features that are strongly affected by extrinsic environmental effects, such as disorder from the substrate and surface additives^10^. Here we provide an investigation on the intrinsic excitonic behaviour in thicker, bulk-like SiP_2_ flakes. Such a tightly bound unconventional exciton in SiP_2_ not only can be envisioned as a platform for the exploration of exciton–phonon (ex–ph) coupling^23–28^ and other many-body physics but also may lend itself to potential applications for anisotropic optoelectronic devices.

## Crystal structure and electronic property of SiP_2_

**Fig. 1:**
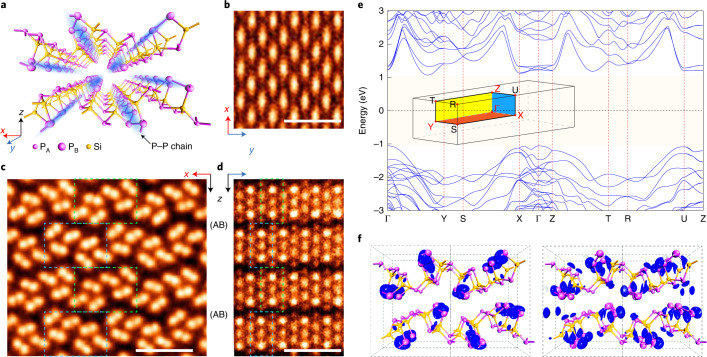
Crystal structure and band structure of layered SiP_2_. **a**, Schematic layered structure of SiP_2_ (*Pnma*, group number 62). The *x,y,z* coordinate system is defined according to the crystal structure, as shown in the bottom-left corner. The blue shading highlights the P_B_–P_B_ chains formed by the P_B_ atoms along the *y* direction of the crystal lattice, which play a critical role in generating quasi-1D electronic and excitonic states. **b**–**d**, Top view (**b**) and cross-sectional (**c**,**d**) STEM–ADF images of SiP_2_ viewed along the *y* axis (**c**) and *x* axis (**d**). Green and cyan dashed rectangles represent the periodic lattice with ABAB stacking order of SiP_2_ layers. Scale bars, 1 nm. **e**, Electronic band structure of bulk SiP_2_ calculated from the *GW* method. The inset shows the first BZ of bulk SiP_2_. SiP_2_ is a semiconductor with an indirect band gap of 2.14 eV. The valence band maximum is at the Γ point, and the conduction band minimum is located along the Γ–Y direction. The conduction band minimum state does not contribute to the formation of the A exciton due to the large direct interband transition energies at this location. **f**, Charge density distribution of the conduction band edge (left) and valence band edge (right) in real space. The isosurface of the plot is 0.02 e Å^−^^3^. Source data

Layered SiP_2_ is chosen as our target material because of its following unique characteristics. Compared with hexagonal layered materials such as graphene and MoS_2_, the cleavable SiP_2_ crystal (space group *Pnma*) possesses an orthorhombic layered structure with a huge in-plane lattice anisotropy, as schematically shown in Fig. 1a and experimentally confirmed by scanning transmission electron microscopy–annular dark-field (STEM–ADF) imaging in Fig. 1b–d and Supplementary Figs. 1 and 2. Remarkably, based on their atomic surroundings, two types of inequivalent phosphorus atoms P_A_ and P_B_ can be distinguished in the SiP_2_ lattice. As shown in Fig. 1a, P_A_ binds to three silicon atoms, while P_B_ binds to one silicon atom and the other two equivalent P_B_ atoms. Note that the P_B_ atoms along the *y* direction of the crystal lattice can naturally form phosphorus–phosphorus chains (denoted as P_B_–P_B_ chains) embedded in the bulk SiP_2_ (blue shades in Fig. 1a), which play a critical role in realizing the quasi-1D electronic states involved in exciton formation. To identify the variation in the chemical bonding environment around P_A_ and P_B_ atoms and the resulting unique properties of P_B_–P_B_ chains in layered SiP_2_, we performed arsenic doping experiments (Supplementary Information, section 2.4) and used STEM characterization (Supplementary Fig. 5). One can see that the doped arsenic atoms only selectively substitute the P_B_ atoms inside the P_B_–P_B_ chains (more details in Supplementary Fig. 5), indicating that the atomic structure containing P_B_–P_B_ chains in SiP_2_ is distinct from the buckled structure in black phosphorus.

More importantly, the anisotropy induced by quasi-1D P_B_–P_B_ chains in layered SiP_2_ directly results in unique electronic properties. Figure 1e shows the band structure of semiconducting bulk SiP_2_ obtained from *GW* calculations. We found that the conduction band edge states in the X–Γ–Z plane of the first Brillouin zone (BZ) are relatively flat with a large effective mass (Supplementary Table 1), and the corresponding charge densities are localized on the P_B_–P_B_ chains (Fig. 1f), behaving like 1D-confined electrons. Importantly, in the direction along the P_B_–P_B_ chains (the *y* direction of the crystal lattice), the electron hopping on P_B_ atoms is significantly larger (bandwidth, ∼1.63 eV) than that across the P_B_–P_B_ chains (bandwidth, ∼0.08 eV) (see details in Supplementary Fig. 22), confirming the 1D nature of this electronic state on the conduction band edge. On the other hand, the hole states at the valence band edge do not show the same level of anisotropy (Supplementary Information, section 2.2), which, compared with 1D electrons, are relatively extended over the whole atomic plane in a quasi-2D fashion. The hybrid dimensionality of these band edge states in SiP_2_ is remarkably different from those of the anisotropic 2D states in black phosphorus^15,29,30^. By analysing the calculated phonon bands given in Supplementary Information, section 13, we identify that the optical phonons localized on P_B_ atoms and neighbouring silicon atoms could have a large coupling with quasi-1D electronic states in layered SiP_2_.

## Exciton with 1D-confined electron and 2D-confined hole

**Fig. 2:**
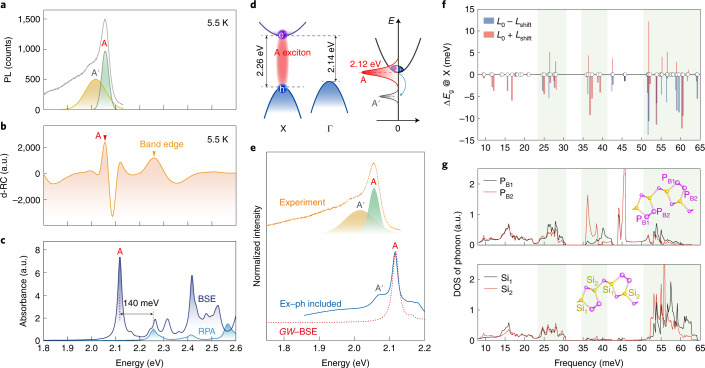
PL, absorption and ab initio calculations of the A exciton and its sideband. **a**, PL spectrum (grey solid line) measured at 5.5 K and its fitted results using two Gaussian peaks plus a background. The fitted peaks are assigned as A (green solid line) and Aʹ (yellow solid line). **b**, The second derivative of the reflectance contrast (d-RC) spectrum (orange solid line) measured at 5.5 K. The arrows indicate the absorption peaks associated with the band edge (yellow) and the A exciton (red). **c**, Calculated absorption spectra of SiP_2_ by using the *GW*–BSE (dark blue) and *GW*–RPA (cyan) methods. The yellow dashed line represents the band-edge transition, the red dashed line represents the A exciton, and the binding energy is 140 meV. **d**, Left: schematic diagram for excitons bound by the Coulomb interaction and electronic band structures for bulk SiP_2_. Right: schematic diagram of the quasiparticle band for excitonic states, which includes the exciton peak A (2.12 eV) and the sideband A′. **e**, Calculated absorption spectrum of SiP_2_ with (blue solid line) and without (red dotted line) ex–ph interactions and the experimental PL spectrum (orange dashed line). The main A exciton peak of the blue line is obtained from *GW*–BSE calculations. The green and yellow shaded Gaussian peaks are A and Aʹ, respectively, as defined in **a**. **f**, Energy shifts of the band gap at the X point $E_{\mathrm{g}}^{{{\mathrm{X}}}}$ individually induced by each optical phonon mode with momentum **q** = 0 at a temperature of zero. *L*_0_ represents the lattice structure without displacement of phonon modes. ±*L*_shift_ stands for atomic displacements of phonon modes. The change in the band gap is estimated by averaging the energy shifts of the band gap $E_{\mathrm{g}}^{{{\mathrm{X}}}}$ with positive (red bar) and negative (dark blue bar) atomic displacements. Black circles denote energies of the corresponding phonon modes. **g**, The phonon density of states (DOS) for optical phonon modes, which is projected to the P_B_ atoms in the embedded P_B_–P_B_ chains (top) and their neighbouring silicon atoms (bottom). Insets: the P_B_ atoms and their neighbouring silicon atoms. Source data

Figure 2a,b presents the PL spectrum and the second derivative of the RC (d-RC; see Supplementary Information, section 6) of an SiP_2_ flake (228 nm) at 5.5 K, which reflects the light emission and absorption properties, respectively. The PL spectrum shows a main peak A at 2.06 eV (the lowest bright excitonic bound state denoted as the A exciton) and a broadened sideband feature Aʹ at 2.01 eV. The main peak A, obtained from all SiP_2_ flakes measured at 5.5 K, is consistently located at an emission energy of 2.06 ± 0.01 eV (here, 0.01 eV is the energy uncertainty obtained from the standard deviation of the emission energies of several measured SiP_2_ flakes; see Supplementary Information, section 7). Such a peak A in the PL spectrum matches the peak at 2.05 eV in the d-RC spectrum, as indicated by the red arrow (Fig. 2b). Due to the interference of the RC signals from the different interfaces in the SiP_2_ thin films supported by substrates (Supplementary Information, section 6), the phonon sideband feature is difficult to identify from the d-RC spectrum.

**Fig. 3:**
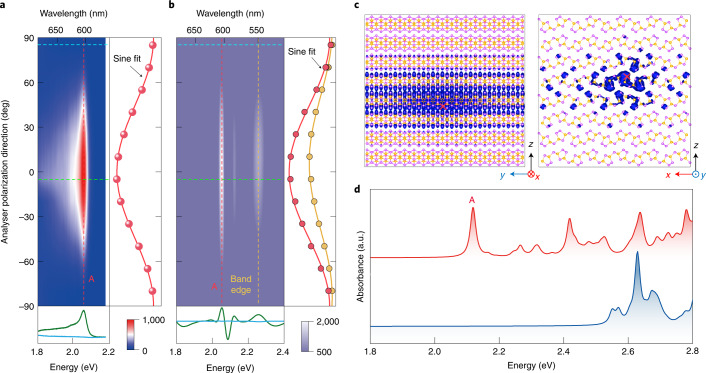
Linear polarized nature of A exciton. **a**, Contour plot of the PL intensity as a function of emission photon energy at different detection polarization angles *θ*, which denotes the angle between the analyser polarization direction and the *x* (defined in Fig. 1a) axis. Right: PL intensity (horizontal axis) and its sine fit versus detection angle (vertical axis) along the red dashed vertical line at 2.06 eV. ‘A’ indicates the position of the A exciton. Bottom: PL spectra with detection polarizations near 90° (cyan line) and 0° (green line) along the corresponding coloured horizontal dashed lines. Colour bar indicates the PL intensity. **b**, Contour plot of the d-RC as a function of photon energy and detection polarization angle. Right: d-RC intensities (horizontal axis; coloured circles) and their sine fits (coloured solid lines) versus the detection angle (vertical axis) along the yellow dashed vertical line at 2.26 eV and red dashed vertical line at 2.05 eV. ‘A’ and ‘Band edge’ indicate the positions of the A exciton and band edge, respectively. Bottom: d-RC spectra with detection polarizations near 90° (cyan line) and 0° (green line) along the corresponding coloured horizontal dashed lines. Colour bar indicates the d-RC intensity. The intensities in the right and bottom panels of (**a**) and (**b**) can be obtained from the contour plots. **c**, The modulus squared of the A exciton’s wavefunction in real space distribution calculated from *GW*–BSE calculations. The red cross marks the position of the hole state. **d**, Simulated absorption spectra from the *GW*–BSE calculation along the *x* (red) and *y* (blue) directions. The absorption peak of the A exciton appears only when the polarization is along the *x* direction with the excitation laser incident along the *z* axis. Source data

Figure 2c shows the absorbance spectra obtained from the *GW*–BSE calculation and *GW* calculation with the random phase approximation (*GW*–RPA). Compared with the calculated absorption spectra based on *GW*–BSE and *GW*–RPA, we confirm that the emission peak A at 2.12 eV originates from the recombination of an excitonic state, in which the electronic states for electrons are quasi-1D and related electronic states for holes are quasi-2D (Fig. 1f). As shown in Fig. 2c,d, the calculated binding energy of such an unconventional exciton is approximately 140 meV (for more details about *GW*–BSE calculations, see Methods and Supplementary Information, section 12). From the modulus squared of the exciton wavefunction in real space shown in Fig. 3c, the observed exciton behaves like a Wannier-type exciton with twofold rotational symmetry, in sharp contrast to 2D excitons in monolayer transition-metal dichalcogenides^13^. More importantly, this exciton is embedded in a bulk layered material with an unusual atomic structure in contrast to those of reported pure 1D excitons in semiconductor nanowires^31^ and CNTs^11,12^, leading to strongly anisotropic Coulomb screening for 1D-confined electrons and 2D-confined holes.

## Anisotropic exciton and exciton–phonon coupling

Since the unconventional A exciton is mainly contributed by electrons and holes localized along the X–Γ–Z direction in the first BZ (Supplementary Information, section 12.2), we use the band edge states at the X point as the representative **k**-point to explore the influence of electron–phonon interactions on its electronic structures and optical response. Figure 2f shows the zero-point energy shifts of the band gap at the X point induced by all optical phonon modes with momentum **q** = 0 at zero temperature. Here, we use the frozen-phonon approximation^32^ to estimate the influence of optical phonon vibrations on the electronic states at the X point (see Supplementary Information, section 13.2 for more details). Since the electron wavefunctions of the A exciton are localized on the P_B_–P_B_ chains, this unconventional exciton couples most strongly to optical phonons, whose vibrational modes are in the X–Γ–Z plane and involve P_B_ atoms and neighbouring silicon atoms (Fig. 2g). These optical phonon modes dramatically modify the electronic structures of the quasi-1D states (Supplementary Information, section 13.4), indicating significant electron–phonon coupling within the P_B_–P_B_ chains. Comparing the results in Fig. 2f,g, one can see that the prominent energy shifts are from the optical phonon modes with eigenenergies of ∼50–60 meV. More details are given in Supplementary Information, sections 13.2 and 13.4.

The experimental observation of the sideband feature Aʹ also indicates that ex–ph interaction on the quasi-1D P_B_–P_B_ chains is at least moderately strong (Supplementary Information, section 8). Therefore, we use a non-perturbative model to simulate the emergence of the sideband feature Aʹ, where a ‘generalized Holstein Hamiltonian’ is used with inputs from first-principles calculations, and the self-energy effects are included beyond the first-order Fan–Migdal diagram (Methods). In this model, we found that the fitted ex–ph coupling constant *M* of 30 meV is comparable to the relatively small bandwidth (or hopping, *t*_ex_ = −20 meV) of the unconventional A exciton (for the estimate of *t*_ex_, see Methods and Supplementary Information, section 13.3). Our approach is similar to the cumulant method considering the ex–ph coupling within the perturbative limit and makes use of the exponential assumption to include the self-energy effects from higher-order diagrammatic terms^33–35^. As shown in Fig. 2e, the appearance of the phonon sideband peak in the simulated spectrum agrees with the experimental results, indicating that sideband Aʹ originates from the ex–ph coupling between the unconventional exciton and the abovementioned optical phonon modes.

Figure 3a,b shows the contour plots of the PL and d-RC intensity of bulk SiP_2_ as a function of emission energy at different detection polarization angles *θ* (*θ* = 0° is set along the *x* direction), suggesting that the linear dichroic absorption and PL emission have similar twofold symmetry characteristics (see Supplementary Information, sections 4 and 12 for more details). Note that the observed linearly polarized PL emission remains along the *x* direction regardless of the incident laser polarization direction or the sample temperature, as shown in Supplementary Figs. 10 and 11. Our *GW*–BSE calculations (Fig. 3d and Supplementary Fig. 21b) show that the absorption peak of the quasi-1D A exciton appears only when the polarization is along the *x* direction. The absorption signal inside the band gap along the *y* direction is forbidden by the SiP_2_ crystal symmetry, which results in relevant optical excitonic matrix elements being zero (Supplementary Information, section 12.3). We also performed pump-probe transient optical measurements to characterize the dynamics of the observed bright exciton in bulk SiP_2_ (see Supplementary Information, section 9 for more details). The lifetime for the exciton in SiP_2_ is as short as 250 fs, which is probably related to an ultrafast process that dissociates these linearly polarized bound excitonic states into unbound and unpolarized states.

**Fig. 4:**
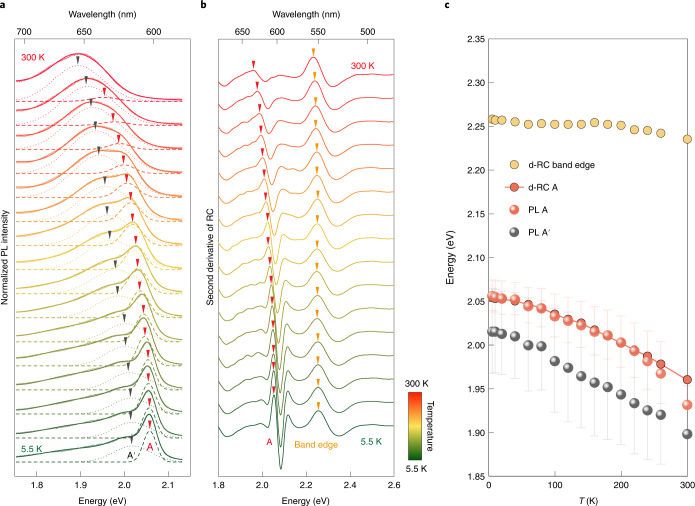
Temperature-dependent spectra and energy evolution for exciton peak A and side peak A′ in SiP_2_. **a**,**b**, Temperature-dependent PL (**a**) and d-RC (**b**) spectra (second derivative of RC) from 5.5 K up to 300 K. The thick solid lines represent the spectra results, while the thin dashed and dotted lines represent fitting results of the PL A and Aʹ peaks. The solid triangle arrows highlight the redshifts of peak A (red), Aʹ (black) and the band edge (yellow) with increasing temperature. **c**, Temperature-dependent band edge (yellow circles) and A exciton (red circles) energies extracted from d-RC spectra, and A exciton (red spheres) and Aʹ (grey spheres) energies extracted by multipeak fitting of PL spectra. The error bars indicates the full width at half maximum. Source data

We further compare the energy shift of the A exciton peak with the energy shift of the quasiparticle band edge as the temperature changes. Figure 4a,b shows the temperature-dependent PL and d-RC spectra for bulk SiP_2_. As shown in Fig. 4c, the optical absorption of the band edges and the exciton peak A, and the sideband feature Aʹ, all exhibit clear redshifts with increasing temperature. The redshifts of the band edge can be fitted with the Bose–Einstein model (see Supplementary Information, section 7 for more details), suggesting that the interaction between electrons and phonons plays an important role in the energy shifts. The redshift of the band edge absorption resulting from the electron–phonon coupling^21,22,36^ is approximately 20 meV at 300 K. On the other hand, the redshift of both peaks A and Aʹ is approximately 90 meV at 300 K, much larger than the energy shift of the direct band edge, indicating an additional contribution from the large coupling between the bound exciton and optical phonons. Such a result is consistent with the analysis of temperature-dependent linewidth broadening of the peak for unconventional A exciton (see Supplementary Information, section 5 for more details).

## Outlook

Using optical spectroscopic measurements with the support of ab initio many-body calculations, we demonstrated the observation of an unconventional bright exciton in layered SiP_2_. In contrast to those reported 1D and 2D excitons truly confined in CNTs and monolayer transition-metal dichalcogenides, the bound excitonic states in layered SiP_2_ exhibit hybrid low dimensionality due to the intrinsic 1D and 2D nature of the constituent electrons and holes, respectively. Interestingly, we envision that SiP_2_ can host peculiar trion states, including a negatively charged trion (composed of two 1D-confined electrons and one 2D-confined hole) and a positively charged trion (composed of one 1D-confined electron and two 2D-confined holes). Once we couple layered SiP_2_ to other vdWs semiconductors, such as monolayer MoS_2_, to form heterostructures, the interfacial layer coupling can change the rotational symmetry of the semiconducting layers and could bring optical and optoelectronic functionalities via symmetry engineering at the heterointerfaces. Through the doping modulation of carrier polarity in SiP_2_ or its heterostructures, rich excitonic physics with exotic dynamic behaviour can be realized in this material platform, such as interlayer excitons and Moiré excitons with tunable dimensionality. Furthermore, the interaction between this unconventional bound exciton and the optical phonon leads to an accompanying phonon sideband. Since a phonon and an exciton fall within the same energy range from zero to several hundred meV, we speculate that such many-body interactions may even lead to the emergence of elementary excitations beyond the Born–Oppenheimer limit in atomic 2D thin films or nanostructures of SiP_2_. Our work will provide a platform to further understand ex–ph coupling and other essential many-body physics and inspire follow-up studies and calculation method developments therein.

## Methods

### SiP_2_ crystal growth with flux method

Single-crystalline samples were synthesized by using the tin flux method^37^. Silicon, phosphorus, gadolinium and tin were mixed at a Si:P:Gd:Sn ratio of 1:6:0.03:5 and sealed into evacuated quartz tubes. The mixture was slowly heated to 1,100 °C to avoid bumping phosphorous and kept for 48 h. Subsequently, the sample was cooled to 400 °C in 140 h and then cooled to room temperature by switching off the electric furnace. The tin flux was removed by using diluted HCl (aq). The obtained black crystals were then ultrasonicated in distilled water and ethanol to remove the residuals (such as phosphorous, adhered) on the crystal surface. This procedure was repeated until the water (or ethanol) became transparent enough after ultrasonication.

### Sample preparation for optical measurements and STEM–ADF measurements

SiP_2_ flakes with thicknesses of 5–200 nm were prepared by mechanical exfoliation onto SiO_2_/Si wafers (300-nm-thick SiO_2_ layer) or fused silica substrate. The thickness was identified by atomic force microscopy (integrated with a WITec Alpha 300 Raman system) after all optical measurements were finished. SiP_2_ is stable in a nitrogen atmosphere and in vacuum and can gradually degrade when exposed to air within several hours. To avoid sample degradation, the whole sample preparation was processed in a glovebox. Atomic-resolution STEM–ADF imaging was performed on an aberration-corrected ARM200F equipped with a cold field-emission gun operating at 80 kV. The STEM–ADF images were collected using a half-angle range from ∼81 to 280 mrad. The convergence semi-angle of the probe was ∼30 mrad.

### Optical measurements

Optical measurements, including the PL spectra and RC and Raman spectra, were performed using a confocal Raman system (WITec Alpha 300). Thickness-dependent PL measurements (Supplementary Information, section 3) were carried out at room temperature using a ×100 objective lens with an incident laser (laser power, 0.2 mW) focused to an ∼1 μm spot. Nitrogen conditions were accomplished by protecting samples using continuous nitrogen gas flow. Low-temperature PL and Raman measurements were performed under vacuum conditions with samples installed in a cryostat (Cryo Instrument of America RC102–CFM microscopy cryostat) using a long working distance ×50 objective lens (laser power, 3 mW). For RC measurement, we recorded the subtracted reflectance of the sample normalized by the reflectance of the substrate, that is, ${{{\mathrm{RC}}}} = {{{\mathrm{1}}}} - \frac{{R_{{{{\mathrm{sample}}}}}}}{{R_{{{{\mathrm{sub}}}}}}}$, where *R*_sample_ represents the reflectance of the SiP_2_ sample on the silicon dioxide or quartz substrate and *R*_sub_ represents the reflectance of the bare substrate.

### First-principles calculations

First-principles density functional theory (DFT) calculations were performed by using the projector-augmented wave (PAW)^38,39^ method implemented in the Vienna Ab initio Simulation Package (VASP)^40^. The energy cut-off for the plane wave basis is set to 500 eV. To test the lattice constants to compare with the experimental value, we used the exchange-correlation functionals of generalized gradient approximation (GGA) with Perdew–Burke–Ernzerhof (PBE) type, local density approximation (LDA), and the PBE functional with vdWs corrections to fully relax the lattice structures. The vdWs interactions were included by using the methods proposed by Dion et al.^41^ with the optB88-vdW functional. We found that lattice constants obtained from the method including the vdWs corrections are closest to the experimental values (Supplementary Information, sections 10 and 11), which are used in the following phonon bands, *GW* and *GW*–BSE calculations. During the lattice relaxations, the force convergence criterion was 10^–3^ eV Å^−1^, and a 9 × 21 × 7 **k**-point mesh was sampled over the BZ. For the self-consistent electronic structure calculations, we set the energy convergence criterion to 10^–6^ eV and the **k**-point mesh to 11 × 25 × 9 over the whole BZ. The phonon spectrum was calculated by the PHONOPY package^42^ in the framework of density functional perturbation theory with the finite-displacement approach, in which a 2 × 4 × 1 supercell was employed.

Using VASP^43^, *GW* calculations^44^ were performed using Kohn–Sham DFT wavefunctions (GGA–PBE) calculated on a 4 × 16 × 4 **k**-point mesh as the initial mean field. The dielectric response function used for the fully frequency-dependent eigenvalue-self-consistent *GW* calculation is summed over 1,240 Kohn–Sham states (corresponding to a 100 eV cut-off). The frequency grid is divided into a dense part ranging from 0 to 13.75 eV and a coarse grid tail ranging from 13.75 to 178.78 eV. The grid sampling is non-uniform with 80 frequency grid points, resulting in step sizes ranging from 0.31 eV in the dense grid up to 46.35 eV in the tail^45^. We use multiple iterations in the *GW* calculation to update the eigenvalues of the Kohn–Sham states when calculating both Green’s function *G* and the screened interaction *W* while keeping the initial Kohn–Sham wavefunctions unchanged. Full convergence was reached after five iterations. This procedure results in better agreement with the experimental results because the standard *G*_0_*W*_0_ approach underestimates the band gap by approximately 220 meV. The maximally localized Wannier functions obtained from the Wannier90 packages^46,47^ were used to plot the *GW* quasiparticle band structure (Fig. 1e). In the construction of the Wannier functions, the *s* and *p* orbitals of both the silicon and phosphorus atoms were used as initial trial wavefunctions. The *GW* quasiparticle energies and Kohn–Sham wavefunctions are used to construct the kernel of the BSE^48,49^. We employed the standard Tamm–Dancoff approximation and included ten conduction bands and ten valence bands during the calculation of the *GW*–BSE Hamiltonian.

### Calculation of the spectral function of the phonon sidebands

To model the spectral function of the phonon sidebands, we solved for the dressed polaron Green’s function of the generalized Holstein Hamiltonian^50,51^, *H* = *H*_0_ + *V*, where $H_0 = \mathop {\sum }\limits_{{{\mathbf{q}}}} \omega _{{{\mathbf{q}}}}b_{{{\mathbf{q}}}}^{\dagger} b_{{{\mathbf{q}}}} + \mathop {\sum }\limits_{{{\mathbf{k}}}} {\it{\epsilon }}_{{{\mathbf{k}}}}c_{{{\mathbf{k}}}}^{\dagger} c_{{{\mathbf{k}}}}$ is the unperturbed single-particle Hamiltonian and $V = \mathop {\sum }\limits_{{{{\mathbf{k}}}},{{{\mathbf{q}}}}} M_{{{{\mathbf{k}}}},{{{\mathbf{q}}}}}c_{{{{\mathbf{k}}}} + {{{\mathbf{q}}}}}^{\dagger} c_{{{\mathbf{k}}}}(b_{{{\mathbf{q}}}} + b_{ - {{{\mathbf{q}}}}}^{\dagger} )$ is the interaction Hamiltonian. For the first term constituting *H*_0_, **q** is the crystal momentum of the phonon, $b_{{{\mathbf{q}}}}^{\dagger}$ and *b*_**q**_ are the phonon creation and annihilation operators, and *ω*_**q**_ (which shall be taken as a constant independent of **q**) is the average phonon energy of the dominant optical branch responsible for ex–ph coupling. For the second term constituting *H*_0_, **k** is the centre-of-mass momentum of the exciton, $c_{{{\mathbf{k}}}}^{\dagger}$ and *c*_**k**_ are the exciton creation and annihilation operators, and $\in$_**k**_ is its energy dispersion. We obtain these values from our *GW* calculations. The interaction Hamiltonian, *V*, represents the ex–ph interaction, with *M*_**k**,**q**_ being the ex–ph coupling matrix element. Since there is only one exciton in the model Hamiltonian, its solution is independent of the statistics of particle^1^. The same solution would be obtained for any fermion or boson, such as electrons (which is more common), as long as the particles are free to move. To construct the generalized Holstein Hamiltonian, we first calculate most of its parameters from first-principles calculations and finally fit the ex–ph coupling matrix elements, *M*_**k**,**q**_ (also to be taken as a constant), to the experimental results.

First, we note that although more than one phonon mode contributes to the renormalization of the band gap, the dominant contributing phonon modes fall within the energy range of 50–60 meV (Fig. 2f and Supplementary Fig. 24). Since the phonon bands that project strongly onto the P_B_–P_B_ chain are relatively flat within the X–Γ–Z plane in reciprocal space (Supplementary Fig. 23b), we assume the representative phonon band to have negligible phonon bandwidth, as in the Einstein model. Hence, we use the energy of a representative longitudinal optical (LO) phonon mode, *ω*_**q**_ ≡ *ω*_LO_ = 55 meV, to model the ex–ph interaction, as obtained from the ab initio phonon calculations. We also assumed that the exciton has a free-particle dispersion of a periodic 1D chain, namely, ${\it{\epsilon }}_{{{\mathbf{k}}}} = - 2t_{{{{\mathrm{ex}}}}}\,{{{\mathrm{cos}}}}(ka)$, where 4*t*_ex_ = −80 meV is the exciton bandwidth. The exciton hopping term, *t*_ex_, was estimated using the hole bandwidth (4*t*_h_ ≈ −80 meV) and the electron bandwidth (4*t*_h_ ≈ 640 meV), which are calculated along the X–Γ–Z direction of the Brillouin zone from our *GW* calculations (Supplementary Fig. 22), with the formula $t_{{{{\mathrm{ex}}}}}^{ - 1} = t_{{{\mathrm{h}}}}^{ - 1} + t_{{{\mathrm{e}}}}^{ - 1}$. Finally, using the above parameters obtained from the first-principles calculations, we only fit the ex–ph parameter, *M*_**k**,**q**_ ≡ *M* = 30 meV, so that the spectral function of the calculated dressed Green’s function reproduces the PL spectrum shown in the optical experiments (Fig. 2e).

In the calculation of the dressed interacting polaron Green’s function, *G* (**k**,*ω*), Dyson’s identity, $\left[{G\left({{{{\mathbf{k}}}},\omega } \right)} \right]^{ - 1} = \left[ {G_0\left({{{{\mathbf{k}}}},\omega } \right)} \right]^{ - 1} - {{{\mathrm{{\Sigma}}}}}({{{\mathbf{k}}}},\omega )$ is used, where *G*_0_(**k**,*ω*) is the free-particle Green’s function, given by $G_0\left({{{{\mathbf{k}}}},\omega } \right) = \left( {\omega - {\it{\epsilon }}_{{{\mathbf{k}}}} + i\eta } \right)^{ - 1}$ and Σ(**k**,*ω*) is the ex–ph self-energy, which consists of an infinite sum of all proper self-energy diagrams. Written more explicitly, *G*(**k**,*ω*) can be written as a continued fraction,

$$\begin{array}{l}{{{{G}}}}\left({{{{\mathbf{k}}}},\omega } \right) = \frac{1}{{G_0^{ - 1}\left( {{{{\mathbf{k}}}},\omega } \right) - \frac{{M^2}}{{G_0^{ - 1}\left( {{{{\mathbf{k}}}},\omega - \omega _0} \right) - \frac{{2M^2}}{{G_0^{ - 1}\left( {{{{\mathbf{k}}}},\omega - 2\omega _0} \right) - \frac{{3M^2}}{{G_0^{ - 1}\left( {{{{\mathbf{k}}}},\omega - 3\omega _0} \right) - \ldots }}}}}}}}\\ \quad \quad \quad = \frac{1}{{G_0^{ - 1}\left( {{{{\mathbf{k}}}},\omega } \right) - {{{\mathrm{{\Sigma}}}}}\left( {{{{\mathbf{k}}}},\omega } \right)}},\end{array}$$

such that Σ(**k**,*ω*) is the second term in the denominator given by

$${{{\mathrm{{\Sigma}}}}}\left({{{{\mathbf{k}}}},\omega } \right) = \frac{{M^2}}{{G_0^{ - 1}\left( {{{{\mathbf{k}}}},\omega - \omega _0} \right) - \frac{{2M^2}}{{G_0^{ - 1}\left( {{{{\mathbf{k}}}},\omega - 2\omega _0} \right) - \frac{{3M^2}}{{G_0^{ - 1}\left( {{{{\mathbf{k}}}},\omega - 3\omega _0} \right) - \ldots }}}}}},$$

that when expanded in powers of *M*^2^ reproduces the Feynman diagrams of each order^52–54^. In the calculation of the self-energy, we used the momentum-averaged non-interacting Green’s function, as introduced by Berciu^53^ and extended by Goodvin, Berciu and Sawatzky^54^. In this approximation, the momentum-dependent non-interacting Green’s function, *G*_0_(**k**,*ω*), in the expression of the self-energy, was replaced by its momentum average, $\bar G_0\left(\omega \right)$, given by $\bar G_0\left(\omega \right) = \frac{1}{{N_{{{\mathbf{k}}}}}}\mathop {\sum }\limits_{{{\mathbf{k}}}} G_0\left( {{{{\mathbf{k}}}},\omega } \right) = \mathop {\smallint }\limits_{ - \infty }^\infty d{\it{\epsilon }}\rho _0\left( {\it{\epsilon }} \right)G_0({\it{\epsilon }},\omega ) = \frac{{{{{\mathrm{sgn}}}}\left( \omega \right)}}{{\sqrt {\left( {\omega + i\eta } \right)^2 - 4t_{xct}^2} }}$, where *N*_**k**_ is the number of **k**-points and $\rho _0({\it{\epsilon }})$ is the density of states. The momentum-averaged self-energy, ${{{\mathrm{{\Sigma}}}}}_{{{{\mathrm{MA}}}}}\left(\omega \right)$, is now momentum independent, and the interacting Green’s function is now ${{{{G}}}}\left({{{{\mathbf{k}}}},\omega } \right) = \frac{1}{{G_0^{ - 1}\left( {{{{\mathbf{k}}}},\omega } \right) - {{{\mathrm{{\Sigma}}}}}_{{{{\mathrm{MA}}}}}\left( \omega \right)}}$. Finally, the spectral function was given by the imaginary part of the interacting Green’s function, the main peak of which is fitted to the excitation energy of excitonic state A as obtained from *GW*–BSE calculations.

## Online content

Any methods, additional references, Nature Research reporting summaries, source data, extended data, supplementary information, acknowledgements, peer review information; details of author contributions and competing interests; and statements of data and code availability are available at 10.1038/s41563-022-01285-3.

## Supplementary information

Supplementary Information Supplementary Figs. 1–26, Discussion and Tables 1–6.

## Data Availability

Source data are provided with this paper. The authors declare that data generated or analysed during this study are provided as source data or included in the Supplementary Information. Further data are available from the corresponding authors upon request. Source data are provided with this paper.

## Source data

Source Data Fig. 1 Statistical Source Data
Source Data Fig. 2 Statistical Source Data
Source Data Fig. 3 Statistical Source Data
Source Data Fig. 4 Statistical Source Data
