# Publisher Correction: Unconventional excitonic states with phonon sidebands in layered silicon diphosphide

**DOI:** 10.1038/s41563-022-01315-0

**Published:** 2022-06-21

**Authors:** Ling Zhou, Junwei Huang, Lukas Windgaetter, Chin Shen Ong, Xiaoxu Zhao, Caorong Zhang, Ming Tang, Zeya Li, Caiyu Qiu, Simone Latini, Yangfan Lu, Di Wu, Huiyang Gou, Andrew T. S. Wee, Hideo Hosono, Steven G. Louie, Peizhe Tang, Angel Rubio, Hongtao Yuan

**Affiliations:** 1 National Laboratory of Solid State Microstructures, Jiangsu Key Laboratory of Artificial Functional Materials, College of Engineering and Applied Sciences, and Collaborative Innovation Center of Advanced Microstructures, Nanjing University, Nanjing, China; 2 Max Planck Institute for the Structure and Dynamics of Matter, Center for Free Electron Laser Science, Hamburg, Germany; 3 Department of Physics, University of California, Berkeley, CA USA; 4 Materials Sciences Division, Lawrence Berkeley National Laboratory, Berkeley, CA USA; 5 School of Materials Science and Engineering, Peking University, Beijing, China; 6 Materials Research Center for Element Strategy, Tokyo Institute of Technology, Yokohama, Japan; 7 College of Materials Science and Engineering, National Engineering Research Center for Magnesium Alloys, Chongqing University, Chongqing, China; 8 Center for High Pressure Science and Technology Advanced Research, Beijing, China; 9 Department of Physics, National University of Singapore, Singapore, Singapore; 10 School of Materials Science and Engineering, Beihang University, Beijing, China; 11 Center for Computational Quantum Physics, Simons Foundation, Flatiron Institute, New York, NY USA

**Keywords:** Two-dimensional materials, Electronic properties and materials, Electronic structure, Optical spectroscopy

Correction to: *Nature Materials* 10.1038/s41563-022-01285-3, published online 16 June 2022

In the version of this article initially published, an additional affiliation was mistakenly listed for Angel Rubio, which has now been removed from the HTML and PDF versions of the article.

