# Supplementary material for: Unconventional excitonic states with phonon sidebands in layered silicon diphosphide
Source: Nat Mater. 2022 Jun 16;21(7):773–8. doi: 10.1038/s41563-022-01285-3 (PMC9242852; doi:10.1038/s41563-022-01285-3)
Supplement: Supplementary file 1 — Supplementary Figs. 1–26, Discussion and Tables 1–6. [file 41563_2022_1285_MOESM1_ESM.pdf]

nature portfolio

https://doi.org/10.1038/s41563-022-01285-3

Supplementary information

Unconventional excitonic states with phonon sidebands in layered silicon diphosphide

In the format provided by the authors and unedited

Supplementary Information for

Unconventional Excitonic States with Phonon Sidebands in Layered Silicon Diphosphide

Ling Zhou[1†], Junwei Huang[1†], Lukas Windgaetter[2†], Chin Shen Ong[3], Xiaoxu Zhao[4], Caorong Zhang[1], Ming Tang[1], Zeya Li[1], Caiyu Qiu[1], Simone Latini[2], Yangfan Lu[5,10], Di Wu[1], Huiyang Gou[6], Andrew T. S. Wee[7], Hideo Hosono[5], Steven G. Louie[3], Peizhe Tang[8,2*], Angel Rubio[2,9*], Hongtao Yuan[1*]

[1]*National Laboratory of Solid State Microstructures, Jiangsu Key Laboratory of Artificial Functional Materials and College of Engineering and Applied Sciences, Nanjing University, Nanjing 210000, China.*

[2]*Max Planck Institute for the Structure and Dynamics of Matter, Center for Free Electron Laser Science, 22761 Hamburg, Germany.*

[3]*Department of Physics, University of California at Berkeley, and Materials Sciences Division, Lawrence Berkeley National Laboratory, Berkeley, California 94720, USA.*

[4]*School of Materials Science and Engineering, Nanyang Technological University, 637371, Singapore.*

[5]*Materials Research Center for Element Strategy, Tokyo Institute of Technology, 4259 Nagatsuta, Midori-ku, Yokohama 226-8503, Japan.*

[6] *Center for High Pressure Science and Technology Advanced Research, Beijing 100094, China.*

[7]*Department of Physics, National University of Singapore, 2 Science Drive 3, 117542, Singapore.*

[8] *School of Materials Science and Engineering, Beihang University, Beijing 100191, China.*

[9]*Center for Computational Quantum Physics, Simons Foundation Flatiron Institute, New York, NY 10010, USA.*

[10]*College of Materials Science and Engineering, National Engineering Research Center for Magnesium Alloys, Chongqing University, Chongqing 400030, China*

E-mail: htyuan@nju.edu.cn; peizhet@buaa.edu.cn; angel.rubio@mpsd.mpg.de

[†] These authors contributed equally to this work.

Outline:

1. **Crystal structure and atomic imaging with STEM of SiP_2**
2. **Identification of the unique quasi-one-dimensional (1D) P_B–P_B chain and unconventional excitonic states in SiP_2**
3. **Optical setup, Raman, and PL spectra of SiP_2**
4. **Linear dichroism of the A exciton**
5. **Temperature-dependent linewidth of the A exciton**
6. **Reflectance contrast spectra and reflectance simulations**
7. **Temperature-dependent energy shifts of the band edge and A exciton**
8. **Comparison of the phonon sideband and exciton–phonon coupling strength**
9. **Pump-probe measurements and linearly polarized transient reflection**
10. **Lattice structures from DFT calculations**
11. **Calculated electronic band structures of bulk SiP_2 based on different exchange-correlation functionals**
12. **Excitons from *GW*–BSE calculations for bulk SiP_2**
13. **Influence on electronic bands by electron–phonon interactions for bulk SiP_2**

References

1. Crystal structure and atomic imaging with STEM of SiP_2

To confirm the crystal structure of silicon diphosphide (SiP_2), we performed scanning transmission electron microscopy–annular dark field (STEM–ADF) measurements on SiP_2 thin flakes. Figure S1a–b shows the schematic layered crystal structure and STEM image of bulk SiP_2. The Si and P atoms within each layer have strong chemical bonding to form buckled two-dimensional (2D) structures, and there is a van der Waals (vdWs) interaction between adjacent layers. Evidence has been provided in Fig. 1 of the Main Text that the SiP_2 we used in our study has the ABAB stacking phase.

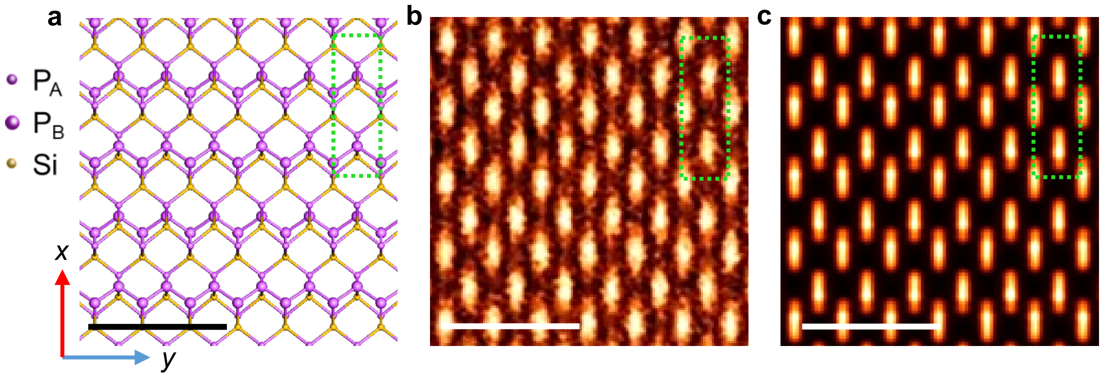

Figure S1 **a**, Schematic crystal structure and **b**, STEM image of SiP_2 with ABAB stacking order, viewed from the *z* direction. Yellow spheres represent Si atoms, and pink spheres represent P_A (small) and P_B (large) atoms. The dashed green rectangles represent the unit cell of SiP_2. **c**, Simulated STEM image viewed from the *z* direction of SiP_2 with ABAB (*Pnma* phase) stacking order. Scale bars stand for 1 nm for all three panels.

To further identify the stacking order of this layered material, we compared the top view of the STEM image with the corresponding STEM simulation shown in Fig. S1c. Such a comparison clearly indicates that the crystal structure in our SiP_2 sample has the *Pnma* space group with ABAB stacking order. The lattice parameters obtained from the STEM results are a = 10.1 Å, b = 3.4 Å, and c = 14.0 Å. To check the uniformity of ABAB stacking in SiP_2 flakes, we performed STEM measurements and obtained large-

area cross-section STEM images on multiple samples or at multiple locations in one sample. Figure S2a–b directly shows the uniform *Pnma* phase with the ABAB stacking order within the SiP_2 crystal. One can see that STEM images, based on randomly chosen areas in the same sample or even on different samples, show the same crystalline features. This fact indicates ABAB stacking within the whole layered SiP_2 bulk crystal.

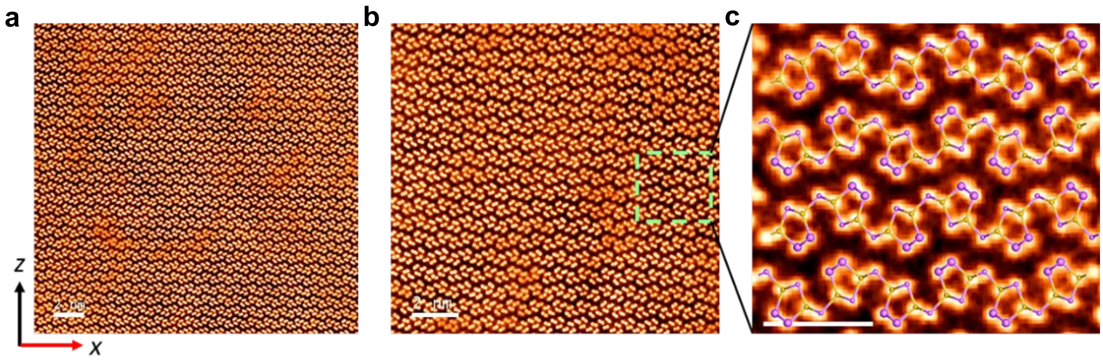

Figure S2 a–b, STEM images of two random areas within the same SiP_2 flake viewed from the *y* direction. Scale bars stand for 2 nm for both images. **c**, Zoomed-in image of the dashed-line green rectangle in panel b and the schematic crystal structure of *Pnma* SiP_2. Scale bar stands for 1 nm.

2. Identification of the unique quasi-one-dimensional (1D) P_B–P_B chain and unconventional excitonic states in SiP_2

As illustrated in Fig. 1a–d in the Main Text, the P_B atoms form quasi-1D chains along the *y* direction, and they are largely different from the P_A atoms. The electrons on the conduction band edges, whose wavefunction is localized on the P_B–P_B chains, clearly show 1D confinement characteristics. The holes on the valence band edges, instead, are slightly more extended within the 2D atomic plane. Herein, we provide additional evidence to support the arguments that the P_B–P_B chains embedded in the SiP_2 bulk crystal play a significant role in the formation of the unconventional excitonic state in SiP_2. The peculiarity of the exciton in SiP_2 highlights how such a quasiparticle is

different from the confined 1D excitonic states in carbon nanotubes (CNTs) whose electrons and holes are both confined in the 1D nanotube and from the anisotropic 2D-like excitons in black phosphorus (BP) whose electrons and holes are both extended in the 2D layers.

2-1 Bader charge analysis for bulk SiP_2

To quantitatively show the chemical environment difference between P_A and P_B, we calculate the Bader charges[1] for all atoms in the unit cell using density functional theory (DFT). As presented in Fig. S3, two kinds of phosphorus atoms (P_A and P_B) in bulk SiP_2 exhibit different chemical properties. For P_A atoms, the Bader charges are 2.0 *e*, suggesting a large charge transfer with the neighboring Si atoms. The chemical bond between P_A and adjacent Si atoms can be classified as an ionic bond. On the other hand, the Bader charge of P_B atoms is 0.2 *e*, indicating that the covalent bond is mainly formed among P_B atoms in the P_B–P_B chains.

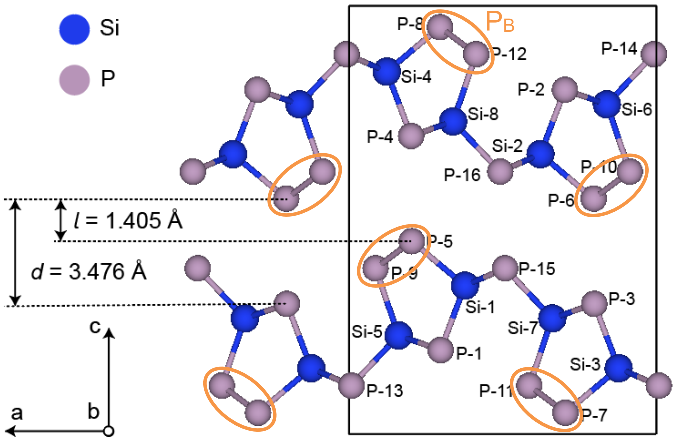

Bader Charge (e)

P_B: P-5 to P-12

Si-1	-2.2e	P-5	0.2e
Si-2	-2.2e	P-6	0.2e
Si-3	-2.2e	P-7	0.2e
Si-4	-2.2e	P-8	0.2e
Si-5	-2.2e	P-9	0.2e
Si-6	-2.2e	P-10	0.2e
Si-7	-2.2e	P-11	0.2e
Si-8	-2.2e	P-12	0.2e
P-1	2.0e	P-13	2.0e
P-2	2.0e	P-14	2.0e
P-3	2.0e	P-15	2.0e
P-4	2.0e	P-16	2.0e

Figure S3 Bader charge analysis for bulk SiP_2. All the atoms in the unit cell are labeled for a clear illustration. There are eight silicon atoms (Si atoms labeled 1–8) and two kinds of P atoms (P atoms labeled 5–12 are P_B atoms marked by orange ellipses, and the remaining P atoms labeled 1–4 and P atoms labeled 13–16 are P_A atoms) in the primitive cell. The Bader charge for all the atoms in SiP_2 are calculated as presented in the right table.

2-2 Comparison of effective masses and bandwidths in anisotropic excitonic systems

To quantitatively highlight the difference between the unconventional excitons in SiP_2 and the previously investigated 1D excitons (e.g., in CNTs[2,3]) and anisotropic excitons (e.g., in BP, ReS_2, and $ReSe_2$[4–6]), we compare the calculated effective masses for the conduction and valence bands and corresponding bandwidths in SiP_2 with those of other anisotropic excitonic materials.

In Table S1, we provide the effective masses for the conduction and valence bands at the X point and the corresponding bandwidths for the band edge states from *GW* calculations in bulk and monolayer SiP_2 and compare them to those parameters in CNTs, BP, ReS_2 and $ReSe_2$. We define the "effective mass/bandwidth anisotropy" as the ratio of effective mass/bandwidth values along two different directions: the direction along the 1D chains (the y direction in the SiP_2 lattice) and the cross-chain direction. One can see that the effective mass anisotropic ratio in bulk SiP_2 is as large as 15.93 for electron states on the conduction band edge, which is larger than that in bulk BP (9.58). For hole states on the valence band edge, the effective mass anisotropic ratio in bulk SiP_2 is 2.37, which is much smaller than those in bulk BP (6.45), bulk ReS_2 (4.29), and bulk $ReSe_2$ (3.66) (see Table S1). In a similar fashion, the conduction bandwidth of bulk SiP_2 in the cross-chain direction is as low as 0.08 eV, while that in the direction along 1D chains is as large as 1.63 eV, giving a bandwidth anisotropic ratio as high as 20 (see Table S1). These results clarify the difference in hopping for electrons and holes along and across the P_B–P_B chains. In contrast, the conduction and valence bandwidths in both directions of BP, ReS_2, and $ReSe_2$ are of the same order of magnitude, suggesting a relatively extended distribution of both electrons and holes on these band edges. Finally, for CNTs, the electrons and holes are fully confined on the nanotube, exhibiting 1D features.

Such exotic electronic properties for the electrons and holes taking part in the formation of excitons in layered SiP_2 result in Coulomb screening, which is different from those in anisotropic 2D layered semiconductors and 1D CNTs. Therefore, we conclude that

the electron–hole pairs bound via the attractive Coulomb interaction should exhibit hybrid dimensional features in layered SiP_2, which are different from the anisotropic excitons in anisotropic 2D layered semiconductors and 1D excitons in CNTs.

2-3 Total charge density of SiP_2 calculated from DFT calculations

Figure S4 shows the calculation results of the total charge density of SiP_2. One can clearly see that the total charge density is mainly contributed from P_B–P_B chains, and the charges surrounding the P_B atoms connect these atoms with each other along the *y* direction and form the 1D P_B–P_B chains of SiP_2.

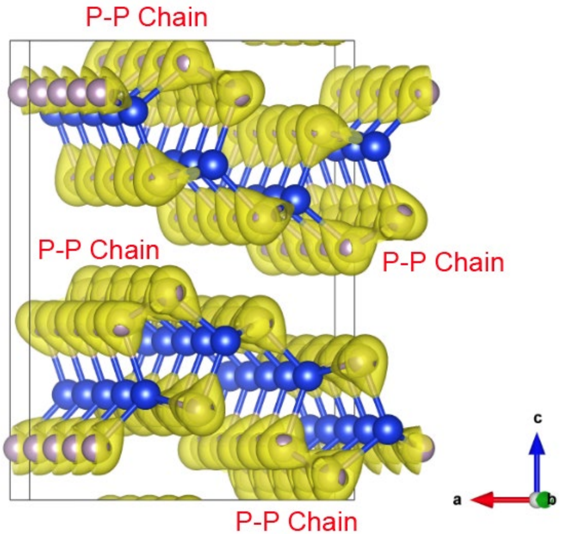

Figure S4 Total charge density of bulk SiP_2 calculated from DFT calculations. From the DFT calculations, we obtain the ground state of bulk SiP_2 and plot the whole charge density for all occupied states. The iso-value is 0.096 e/Å^3.

Table S1. Comparison of the anisotropic effective mass and bandwidth in layered materials

Materials		Effective mass (m_0)			Bandwidth (eV)			Reference
		Along-chain	Cross-chain	Anisotropic ratio	Along-chain	Cross-chain	Anisotropic ratio	
Bulk SiP_2	*e*	0.384	6.121	15.93	1.63 (X–S)	0.08	20	This work
Bulk SiP_2	*h*	−1.555	−3.683	2.37	1.37 (X–S)	0.64	2	This work
1L-SiP_2	*e*	0.419	14.677	35.03	NA	NA	NA	This work
1L-SiP_2	*h*	−2.324	−12.057	5.19	NA	NA	NA	This work
(5,1) SWCNT	*e*	0.159	NA	∞	NA	NA	NA	Ref. 7
1L-BP	*e*	0.17	1.12	6.59	1.13 (Γ–Y)	1.35 (Γ–X)	1.19	Ref. 8
1L-BP	*h*	−0.15	−6.35	42.33	2.13 (Γ–Y)	~ 3 (Γ–X)	1.41	Ref. 8
Bulk BP	*e*	0.12	1.15	9.58	NA	NA	NA	Ref. 8
Bulk BP	*h*	−0.11	−0.71	6.45	NA	NA	NA	Ref. 8
1L-$ReSe_2$	*e*	NA	NA	NA	0.11 (Γ–K3)	0.11 (Γ–M1)	1	Ref. 9
1L-$ReSe_2$	*h*	NA	NA	NA	0.45 (Γ–K3)	0.06 (Γ–M1)	7.5	Ref. 9
Bulk $ReSe_2$	*h*	−1.13	−4.14	3.66	NA	NA	NA	Ref. 10
1L-ReS_2	*e*	NA	NA	NA	0.24 (Γ–K3)	0.32 (Γ–M1)	1.33	Ref. 9
1L-ReS_2	*h*	NA	NA	NA	0.36 (Γ–K3)	0.08 (Γ–M1)	4.5	Ref. 9
Bulk ReS_2	*h*	−1.08	−4.63	4.29	NA	NA	NA	Ref. 10

2-4 Selective substitution of dopant arsenic to P_B atoms in SiP_2

To compare the unique structural properties in the quasi-1D P_B–P_B chain in SiP_2 to the buckled P–P structure in BP, we grew arsenic-doped SiP_2 ($SiP_{2-x}As_x$ alloy) and As-doped BP ($P_{1-x}As_x$ alloy) samples. With STEM characterization, we clearly identified a unique P_B–P_B chain where the substitutional sites of dopant As are located in $SiP_{2-x}As_x$. Figure S5a and c shows schematic side views of the crystal structure of SiP_2 and BP. One can clearly see that there are two types of P atoms in SiP_2, where P_B forms 1D P_B–P_B chains along the *y* direction. In contrast, all P atoms in layered BP are equivalent, and all the anisotropic properties come from the buckled structure of its anisotropic lattice. Figure S5b shows the STEM characterization of $SiP_{2-x}As_x$ viewing along the [010] direction. Compared with the results of SiP_2 shown in the Main Text and Fig. S2, selective replacement of the P atoms at the P_B position with As atoms in $SiP_{2-x}As_x$ crystal structure can be observed, where only P_B atoms in the quasi-1D chain are replaced by As atoms (brighter atoms marked by ellipses in panel b). For the $P_{1-x}As_x$ samples (Fig. S5d), As dopants are homogenous in the crystal lattice, and no preferred dopant site is observed compared to the BP case in our observation and other report[11]. The selective replacement of As atoms with P_B atoms in the quasi-1D chains confirms the unique chemical properties of such a P_B–P_B chain in SiP_2. The totally different substitutional behaviour of As doping in SiP_2 and BP indicates that the quasi-1D P_B–P_B chains in SiP_2 are intrinsically distinct from the structure in BP.

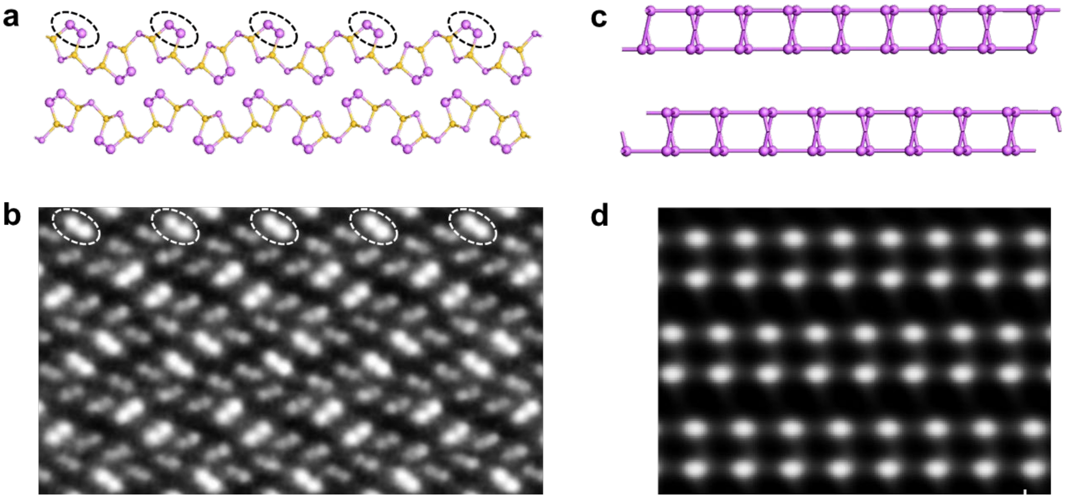

Figure S5 a, Side view of the schematic crystal structure of SiP_2. **b**, STEM characterization results of $SiP_{2-x}As_x$ (As-doped SiP_2) viewing along [010]. The ellipse circles in panels **a** and **b** indicate the quasi-1D P_B–P_B chain locations in SiP_2. **c**, Side view of the schematic crystal structure of BP along [110]. **d**, STEM characterization results of black $P_{1-x}As_x$ viewing along [110].

3. Optical setup, Raman and PL spectra of SiP_2

3-1 Optical setup, Raman and PL spectra

The experimental setup for optical measurements is schematically shown in Fig. S6a. A continuous wave laser (532 nm) was used for the Raman and photoluminescence (PL) measurements, and a white-light LED lamp was chosen in the reflectance measurement. A half-wave plate is used to rotate the polarization direction of the incident laser (excitation polarization). A polarization analyser is used to detect the linear dichroism of the reflected signal from SiP_2 flakes (detection polarization). As mentioned in the Methods section of the Main Text, a cryostat with liquid nitrogen or liquid helium as the cooling agent is used to provide a low-temperature measurement platform down to 4.4 K. Figure S6b shows typical Raman spectra of bulk SiP_2. Our measurement (red line) is in good agreement with the previous Raman study (black line) on SiP_2 with the *Pnma* structure[12] and directly confirms the ABAB stacking sequence of the SiP_2 crystal.

Figure S6c shows the linear dichroic polarization-resolved PL spectra. The SiP_2 flake with a thickness of 240 nm is intentionally selected and regarded to be thick enough to reflect the typical bulk behaviour of SiP_2. The measurement geometry for linear dichroic PL is given as follows: the excitation polarization is fixed along the *x* direction, and the detection polarization is selectively oriented along either the *x* (noted as *xx*, red line) or *y* (noted as *xy*, dark blue line) directions. Among the three observed PL emission peaks (referred to as peaks A, B and C) in Fig. S6c, peak A shows linear dichroism that is linearly polarized along the *x* direction rather than almost isotropic behaviour in the low-energy PL emissions for peaks B and C. These two peaks might result from the emission of defect energy levels in bulk SiP_2 (discussed later).

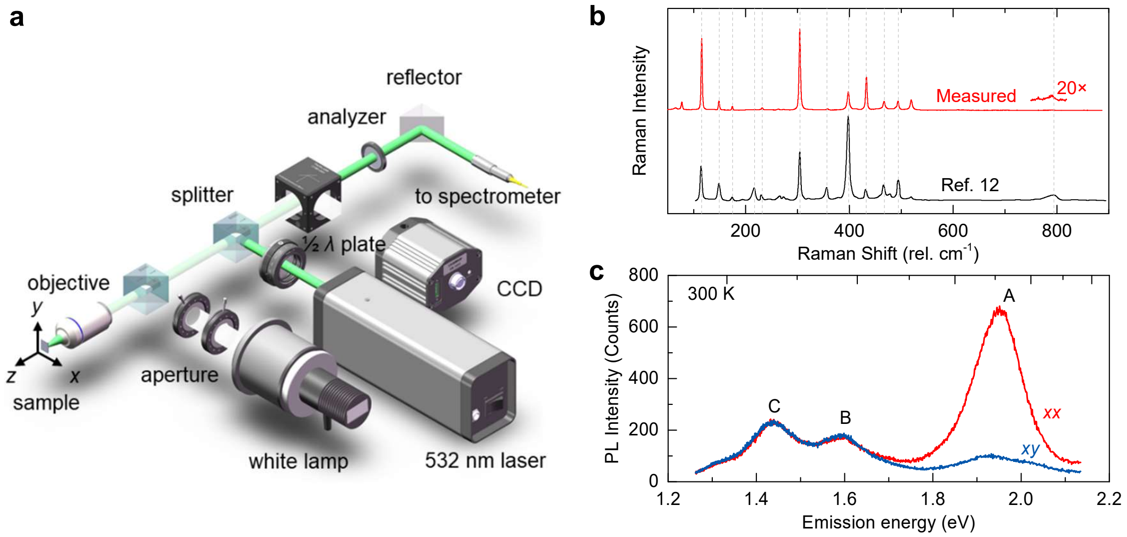

Figure S6 a, Optical setup for spectroscopy measurement. **b**, Raman spectra of bulk SiP_2 crystal measured at 300 K (red line) and the result from ref. 12 (black line). **c**, Typical PL spectra of a SiP_2 flake with a thickness of 240 nm (reflecting the intrinsic property of bulk SiP_2 crystal) measured at 300 K, with excitation polarization along the *x* direction and detection polarization along the *x* (red) and *y* (blue, along the P_B–P_B chain) directions.

3-2 Thickness-dependent and excitation power-dependent PL spectra of SiP_2

Figure S7a shows the thickness-dependent PL spectra on an individual flake with different thicknesses. In the thicker area (60.7 nm) of the flake, all three peaks can be

clearly observed in the PL spectrum, while only peak A remains distinct in the area with a thin thickness (7.4 nm). The thickness-dependent intensities of peaks B and C, normalized by the intensity of peak A, are shown in Fig. S7b. The emission intensity of the defect state is always proportional to the total number of defects inside the flakes and can thus be strongly dependent on the sample thickness. The disappearance of peaks B and C with decreasing thickness provides direct evidence that the B and C emissions are related to the defect state in SiP_2 flakes[13]. Since this is not our main focus associated with the excitonic state of the A exciton and its sideband, the B and C emissions will not be discussed in detail in this study.

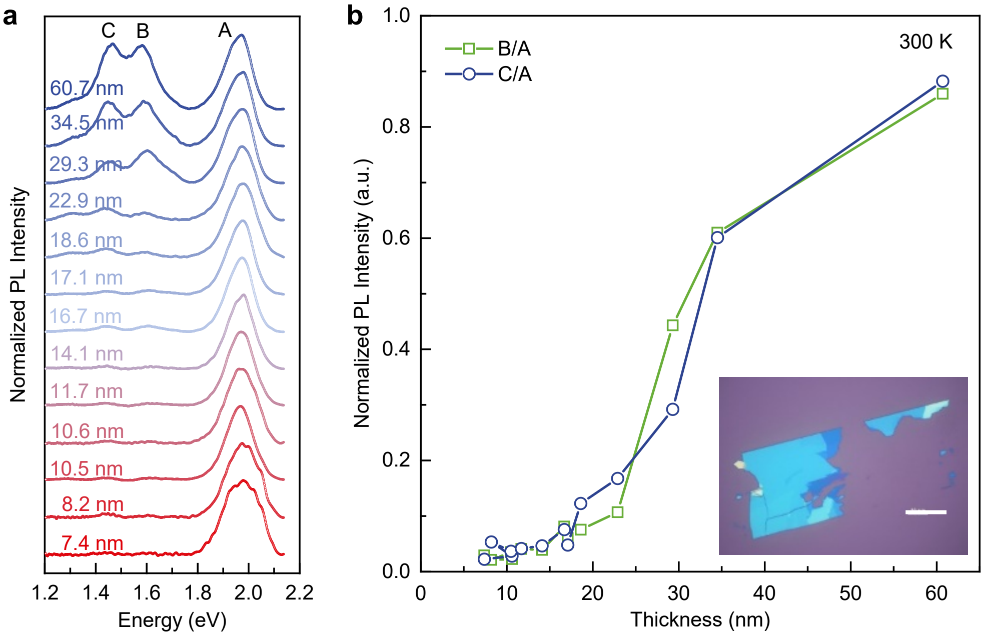

Figure S7 a, Thickness-dependent PL spectra at 300 K. Note that the B and C peaks start to vanish as the sample thickness decreases from 60.7 nm to 7.4 nm. **b**, PL intensity of peaks B and C as a function of sample thickness. PL spectra are normalized by the intensity of peak A. The inset is the optical photograph of the measured samples. Scale bar stands for 20 μm.

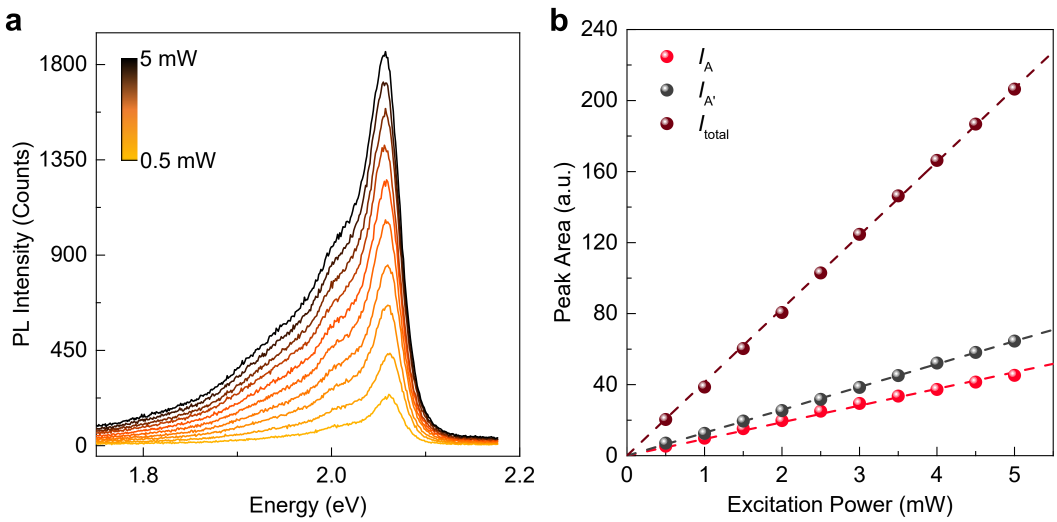

Figure S8 a, Excitation power-dependent PL spectra of a typical SiP_2 sample at 77 K. The colour bar indicates the laser power for the spectra. **b**, Excitation power-dependent peak area of the A exciton (red), A′ sideband (black), and A_{total} (brown, I_{total} is defined as the total peak area in the energy range from 1.8 eV to 2.1 eV). The dashed lines are the linear fitting results to the corresponding data.

Figure S8a shows the PL intensity as a function of excitation laser power. Figure S8b shows the linear dependency of the integrated PL peak area for both exciton A and sideband A′ with an excitation laser power up to 5 mW. As mentioned in the Methods section, the laser power used in PL measurement is always kept within the linearly dependent range, ensuring that no effects from laser heating or nonlinear optical phenomena play any significant role in our obtained spectra[14,15].

3-3 Comparison of the PL intensity of SiP_2 to other 2D materials

To clearly understand the relative PL efficiency of SiP_2, we compared the PL spectra of thick SiP_2 with 1-layer (1L) MoS_2, 2-layer (2L) MoS_2, thick MoS_2, thick ReS_2 and thick $ReSe_2$ in Fig. S9. All spectra were measured under the same conditions. One can clearly see that the PL intensity of thick SiP_2 is approximately 2 ~ 3 orders of magnitude

smaller than that of 1L MoS_2 owing to the indirect band gap nature of bulk SiP_2. However, compared to other anisotropic layered materials, the PL intensity of thick SiP_2 is 10 times larger than that of $ReSe_2$ and is comparable to that of ReS_2.

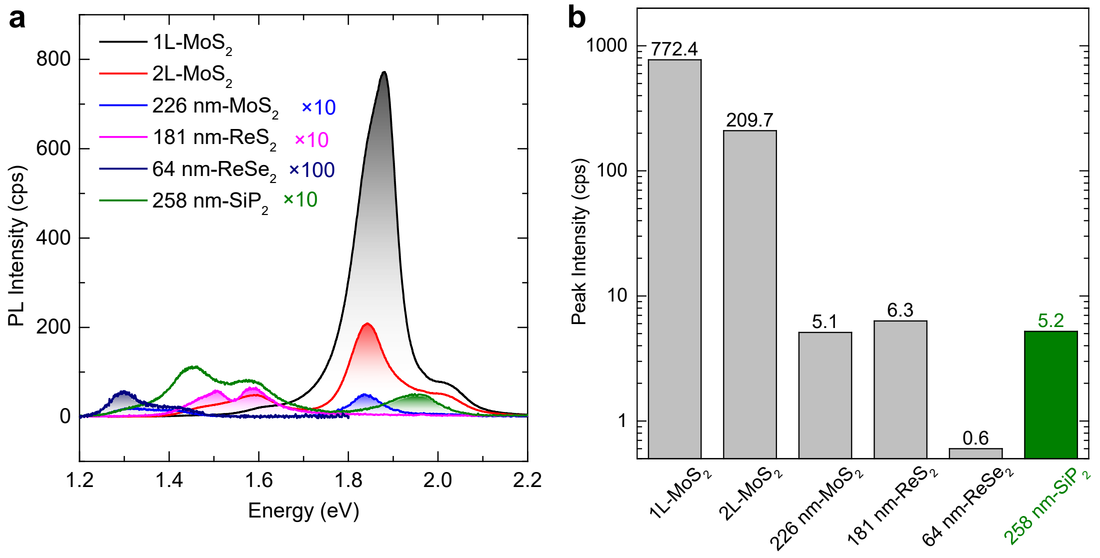

Figure S9 Comparison of the PL efficiency of typical layered materials. **a**, PL spectra and **b**, the peak intensity of the main characteristic peak of typical layered materials, including thick SiP_2 (258 nm), 1-layer (1L) MoS_2, 2-layer (2L) MoS_2, thick MoS_2 (226 nm), thick ReS_2 (181 nm) and thick $ReSe_2$ (64 nm). All PL measurements were performed under the same optical conditions at room temperature with an excitation laser (532 nm) power of 0.2 mW and a spot size of approximately 1 μm^2.

4. Linear dichroism of the A exciton

4-1 Polarization-dependent PL with different excitation polarizations

Figure S10a–c presents the colour plots of the PL intensity as a function of emission energy and the detection polarization angle θ (defined as the angle between the detection polarization direction and the x-axis of the crystal lattice). The excitation polarization is separately set along 0° (Fig. S10a), 45° (Fig. S10b), and 90° (Fig. S10c),

while the photon energy (2.33 eV) and laser power remain unchanged. Figure S10d shows the PL intensity of peak A as a function of the detection polarization angle when fixing the excitation polarization angles to 0° (*x* direction), 45°, and 90° (*y* direction). The solid lines are the fitting results using $\cos^2(\theta)$ functions. From the twofold symmetry of the fitted curves and the observed maximum intensities shown at $\theta = 0°$, we can conclude that the linearly polarized PL emission is fixed along the *x* direction of the crystal regardless of the excitation polarization of the laser. Interestingly, the PL intensity is always stronger with the excitation direction along the *x*-axis than with the excitation direction along the *y*-axis (along the P_B–P_B chain) when fixing the detection polarization. This indicates the anisotropic absorption of the excitation laser of the SiP_2 crystal, which is associated with its anisotropic lattice[4] and consistent with the theoretical calculation results shown in Fig. 3d in the Main Text and Fig. S21.

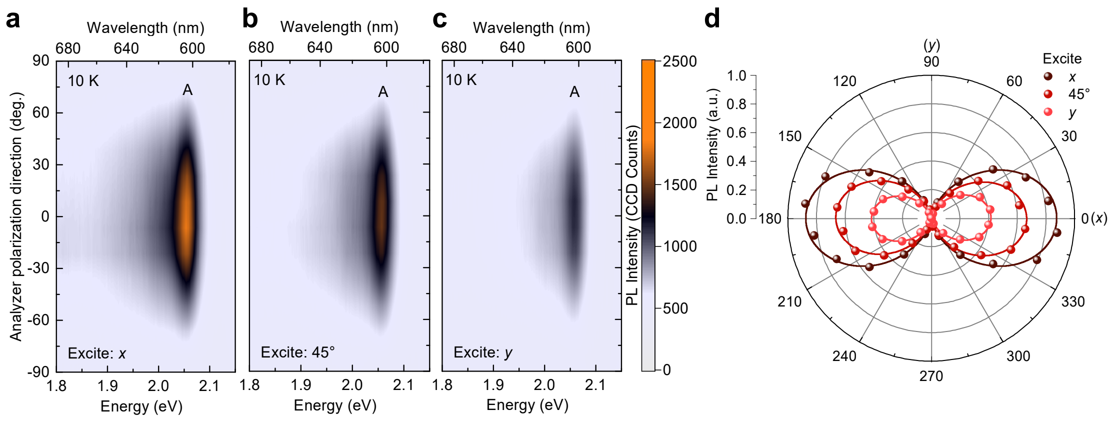

Figure S10 Colour plots of PL spectra as a function of detection polarization angle θ (denotes the angle between the detection polarization and *x*-axis). The excitation polarizations are set along **a**, 0° (the *x* direction of the crystal lattice), **b**, 45° and **c**, 90° (the *y* direction of the crystal lattice, also along the P_B–P_B chain). **d**, Polar plot of PL intensities of peak A as a function of detection angle with excitation polarization along the 0° (brown), 45° (dark red) and 90° (red) directions. The excitation laser power remains unchanged. The solid curves are the fitting results using $\cos^2(\theta)$ functions.

4-2 Polarization-dependent PL at different temperatures

Figure S11 shows the PL peak areas as functions of the detection polarization angle θ at various temperatures down to 4.4 K. The integrated areas of the A exciton peak can be well fitted using $\cos^2(\theta)$ functions and show a linear dichroic nature in the PL emissions. In addition, the linear polarization is maintained along the same direction (the x direction of the crystal lattice) over the whole temperature range.

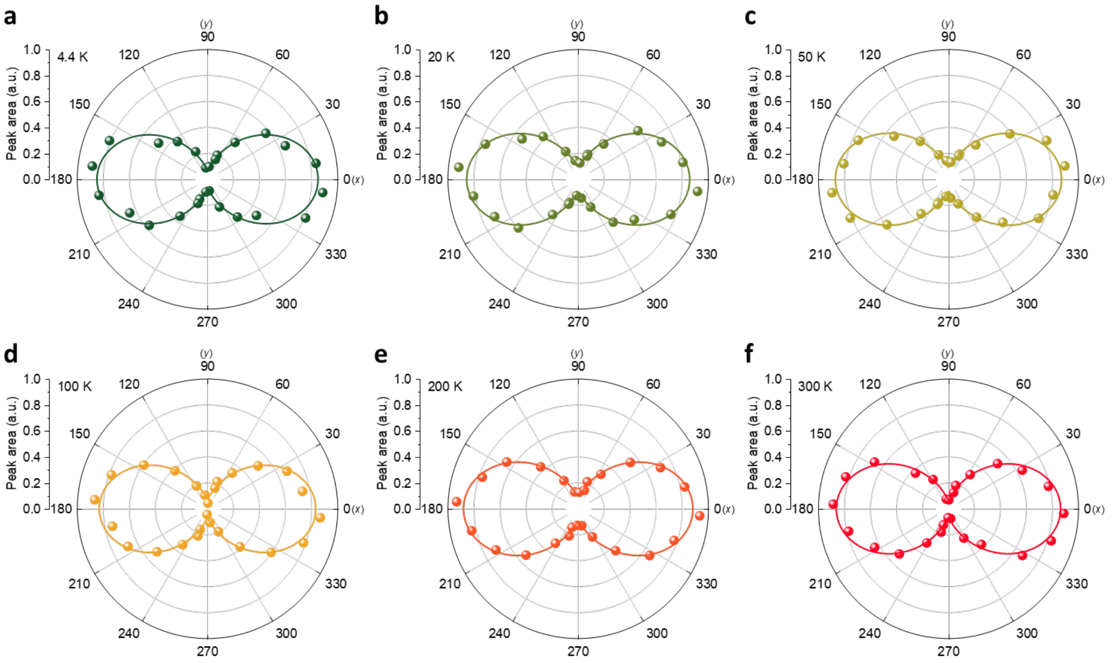

Figure S11 Polar plots of the integrated areas of the A exciton peak as functions of detection angle θ at various temperatures: **a**, 4.4 K, **b**, 20 K, **c**, 50 K, **d**, 100 K, **e**, 200 K and **f**, 300 K.

5. Temperature-dependent linewidth of the A exciton

Previous reports on the excitonic states in transition-metal dichalcogenides (TMDs) have shown that the temperature dependence of the exciton linewidth (denoted as the full width at half maximum of the PL peak) can be described using a general equation[16–19]

$$\gamma(T) = \gamma(0) + c_1 T + \frac{c_2}{e^{E_0/k_B T} - 1} \quad (1)$$

Here, $\gamma(0)$ is the zero-temperature linewidth that is not related to the exciton–phonon (ex–ph) interaction, the linear T term is associated with the exciton–acoustic phonon scattering, and the last term results from the interaction of excitons with optical phonons as well as acoustic phonons on the edges of the first Brillouin zone (BZ) (E_0 is the effective phonon energy, T is the temperature, k_B is the Boltzmann constant, and c_1 and c_2 are related to the ex–ph coupling strength).

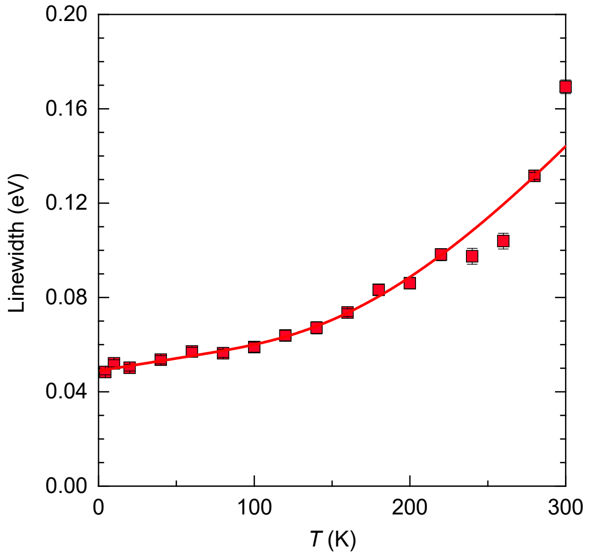

Figure S12 Temperature-dependent linewidth of the PL peak for the A exciton and the fitting result (red line) for exciton linewidth broadening based on equation (1).

Figure S12 shows the temperature-dependent broadening of the exciton linewidth, which can be well fitted using equation (1). The temperature-dependent linewidth broadening is proportional to the effective Bose–Einstein distribution, which indicates that the ex–ph coupling in bulk SiP_2 can play an important role in determining excitonic properties[17,20–22]. Such results are consistent with the observation of the large temperature-dependent redshifts of the A exciton and sideband peak A′. The PL peak

broadens due to the finite lifetime of the excitonic states, and these states are normally influenced by the many-body interactions between excitons and phonons. Thus, at a higher temperature, the broadening is larger, and the ex–ph interaction is stronger because more phonons will be excited at a higher temperature.

6. Reflectance contrast spectra and reflectance simulations

6-1 Substrate-dependent reflectance contrast spectra

To determine the band gap absorption of SiP_2, we performed reflectance contrast (RC) measurements, a classic method to probe the band edge information and excitonic states of semiconductors. Since our samples are directly exfoliated onto the silicon substrate covered by 300 nm SiO_2 (schematically shown in Fig. S14a), Fabry–Pérot (F–P) interferences originating from either the SiO_2 layer[23–25] or sample flakes can always lead to a large interference-type background signal in the RC spectrum. Previous RC spectrum studies on the excitonic states in monolayer TMDs demonstrated that the interference background had some chance to be largely weakened by using a silicon substrate with a thinner SiO_2 layer or transparent substrates[23,25–27]. However, as we have shown in the PL and RC spectra (Fig. S13) of SiP_2 flakes cleaved onto the SiO_2/Si substrate covered with thin SiO_2 (20 nm, 50 nm) or directly on a quartz substrate, one can still see that a remarkable interference background exists in the RC spectra owing to the F–P interference from the SiP_2 flakes with a thickness up to a few hundred nanometers. These results indicate that changing substrates cannot be an effective way to dramatically weaken the F–P interference background. Therefore, we performed an RC simulation to obtain a better understanding of those RC features originating from the intrinsic properties of SiP_2.

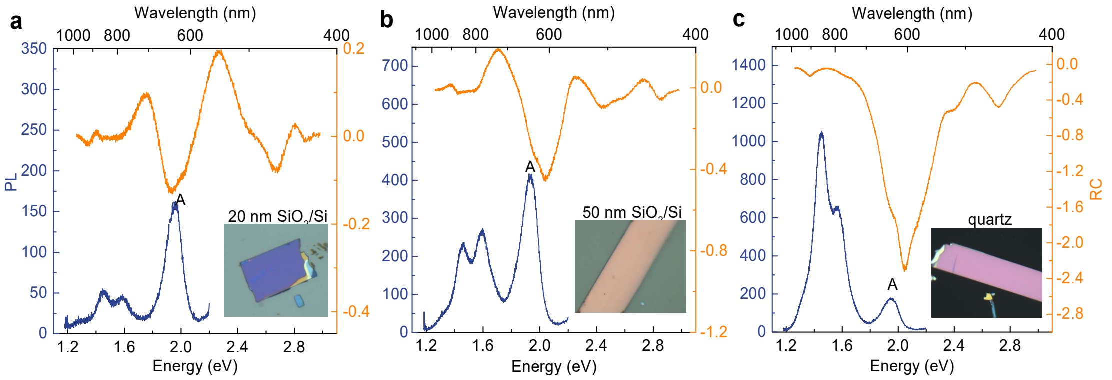

Figure S13 Substrate-dependent PL (dark blue line) and RC (orange line) spectra of SiP_2 measured at 300 K **a**, on a silicon wafer covered with 20 nm SiO_2; **b**, on a silicon wafer covered with 50 nm SiO_2; and **c**, on a quartz substrate. One can clearly see the existence of the F–P interference background in the RC spectra not only on the SiO_2/Si substrate but also on the quartz substrate. The insets are optical photographs of the measured samples.

6-2 Simulation of reflectance contrast spectra

To obtain a more intuitive understanding of the influence of F–P interference on the observed RC spectra, we performed simulations for RC spectra based on the structure shown in Fig. S14a. We use a SiP_2 flake cleaved onto the SiO_2/Si substrate (300-nm SiO_2 layer) as the modeling structure. When white light is illuminated on this system, F–P interference occurs due to the multiple reflection processes at the interfaces of air/SiP_2, SiP_2/SiO_2 and SiO_2/Si. Based on the Fresnel law, we calculate the reflectance of the sample (R_{sample}) and substrate (R_{sub}). Thus, we get reflectance contrast based on $\mathrm{RC} = 1 - \frac{R_{\mathrm{sample}}}{R_{\mathrm{sub}}}$. We list the corresponding formulas and parameters for determining the RC spectra in Tables S2 and S3 (refs. 23,25,28,29).

Figure S14b shows the simulated RC spectra based on the Fresnel law that has been widely used to analyse the reflection spectra for TMDs[25]. It is well known that the dielectric response and the resulting light absorption in condensed matter can be simply modeled using a Lorentz oscillator, whose resonance energy is equal to the photon energy[23,29,30]. The simulated RC spectra are shown with a black line in Fig. S14b. In

this case, we regard the sample as a dielectric material without considering any light absorption related to optical transitions. As a direct result, the transparent SiP_2 sample contributes to the interference, similar to the SiO_2 layer. For the simulated RC spectra shown by the red line in Fig. S14b, in addition to the abovementioned interference, we also take SiP_2's light absorption into account the optical transition (described by a Lorentz oscillator). Two important features of the reflectance (red line, after considering the light absorption) need to be addressed here. First, the broad dip at approximately 2.1 eV is almost the same as that shown by the black line, which indicates that the change in the reflectance signal is induced by F–P interference. Due to the F–P interference-induced dip at 2.1 eV for a sample of typical 200 nm thickness, the sideband feature cannot be distinguished in the RC or d-RC spectra. Second, the absorption dip at 2.01 eV (Here, the resonance energy of the oscillator we used for simulation is centered at 2 eV, see Table S3) can be observed when we consider the contribution from the oscillator to the dielectric constant of SiP_2. The consistency of the dip at 2.01 eV with the resonance energy (2 eV) shows that this dip resulted from the optical transition-related absorption of SiP_2.

This analysis and comparison explain why our RC simulation is scientifically reliable and technically available to help us identify the signal from the A exciton in our RC measurement, even though there is a small but widely accepted inconsistency between the resonance energy used in our simulation and the absorption dip energy obtained from the simulated RC spectra. The RC simulation provides us with a technical tool to distinguish the small absorption signal in RC spectra.

Table S2. Equations and physical parameters used for the RC simulations

Equations	Parameters	Physical Meanings
$\varepsilon(E) = \varepsilon_\infty + \sum_j \frac{E_{pj}^2}{E_{oj}^2 - E^2 - i\gamma_j E}$	ε_∞	high-frequency dielectric constant
	E_{pj}	plasma energy of *j*th oscillator
	E_{oj}	resonance energy of *j*th oscillator
	γ_j	linewidth of *j*th oscillator
$\tilde{n}_i = \sqrt{\varepsilon}$	$\tilde{n}_i$	refractive index of *i*th layer
$\Phi_i = \frac{2\pi\tilde{n}_i d_i}{\lambda(E)}$	Φ_i	phase delay of *i*th layer
	d_i	thickness of *i*th layer
$r_{ij} = \frac{\tilde{n}_i - \tilde{n}_j}{\tilde{n}_i + \tilde{n}_j}$	r_{ij}	reflective of *i*th and *j*th layer interface
$R_{\text{sub}} = I_0 \left\| \frac{r_{02} + r_{23} e^{-2i\Phi_2}}{1 + r_{02} r_{23} e^{-2i\Phi_2}} \right\|^2$		
$R_{\text{sample}} = I_0 \left\| \frac{r_{01} e^{i(\Phi_1+\Phi_2)} + r_{12} e^{-i(\Phi_1-\Phi_2)} + r_{23} e^{-i(\Phi_1+\Phi_2)} + r_{01} r_{12} r_{23} e^{i(\Phi_1-\Phi_2)}}{e^{i(\Phi_1+\Phi_2)} + r_{01} r_{12} e^{-i(\Phi_1-\Phi_2)} + r_{01} r_{23} e^{-i(\Phi_1+\Phi_2)} + r_{12} r_{23} e^{i(\Phi_1-\Phi_2)}} \right\|^2$		
$\text{RC} = 1 - \frac{R_{\text{sample}}}{R_{\text{sub}}}$	RC	reflectance contrast
	R_{sample}	reflectance of sample
	R_{sub}	reflectance of bare substrate

Table S3. The material parameters used in our RC simulations

d_{SiO_2}	d_{sample}	$\tilde{n}_{\text{air}}$	$\tilde{n}_{SiO_2}$	$\tilde{n}_{\text{Si}}$	ε_∞	E_{pj}	E_{oj}	γ_j
300 nm	200 nm	1	1.46	3.61	6.46	0.28 eV	2 eV	0.07 eV

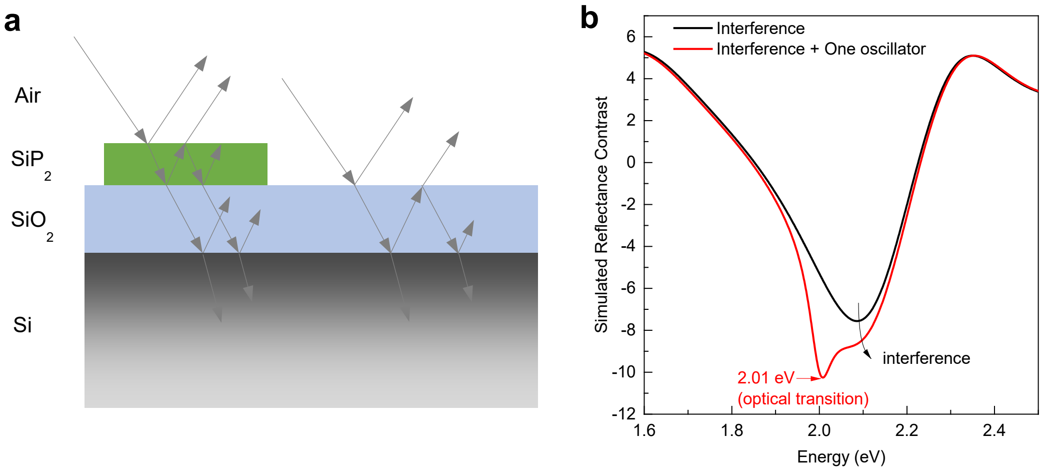

Figure S14 a, Schematic diagram of a model system with a SiP_2 flake cleaved onto SiO_2/Si to simulate the influence of F–P interference on the reflectance. **b**, Simulated RC spectra of SiP_2 on a SiO_2/Si substrate. The black curve shows the RC interference background without considering the absorption of SiP_2, and the red curve shows the RC spectrum considering the SiP_2 absorption. In the red curve, there are two dip features: one corresponds to the optical transition (marked using a red arrow at approximately 2.01 eV), and the other is due to the F–P interference (marked using a black arrow at approximately 2.1 eV). For the RC simulations, the applied simulation parameters are listed in Table S3.

6-3 Linearly polarized reflectance contrast spectra

To understand the light absorption of the excitonic states and band edge information in SiP_2, we carried out polarization-resolved RC measurements. As we mentioned in the Main Text and Sec. 4 in SI, both the absorption and PL emission spectra of SiP_2 show linearly dichroic properties with respect to the polarization of the excitation. As the absorption features in RC spectra change systematically with the detection polarization, the interference background should not show any change in the RC spectra. If we subtract all RC spectra by the spectrum at the detection polarization along the *y* direction (also along the P_B–P_B chain), this will directly provide us with another way to remove the interference background and distinguish the small RC features from the intrinsic property of SiP_2.

Figure S15a presents the RC spectra with different detection polarization angles. The polarization angle is defined as the angle between the analyser and *x* direction of the SiP_2 lattice. As illustrated in Fig. 2a of the Main Text, the A exciton has an emission energy of 2.06 eV, falling in the range of the interference dip. There are two small absorption dips in Fig. S15a: one dip at 2.05 eV corresponds to the A exciton of SiP_2, and the other dip at 2.1 eV is due to the abovementioned F–P interference, marked with red and gray dashed lines, respectively. Note that the dip of 2.05 eV shows the same linear polarization character as that in the PL emission of the A exciton when we change the detection polarization from the *y* direction (± 90°) to the *x* direction (0°). The other feature at approximately 2.26 eV (see the dip at 0°, marked with a light yellow rectangle) is regarded as the direct absorption of band edges.

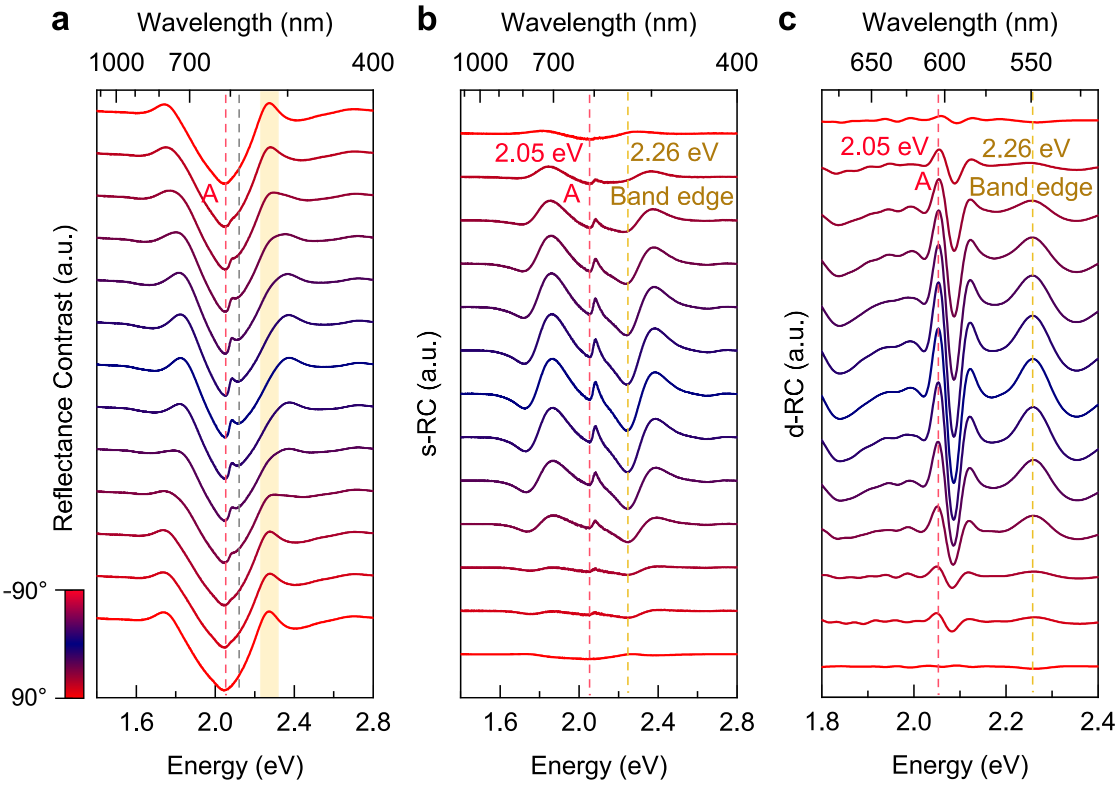

Figure S15 Detection-polarization-resolved RC spectra of SiP_2 obtained at 5.5 K: **a**, RC, **b**, interference background-subtracted RC ($\text{s-RC} = \text{RC} - \text{RC}_{\pm 90^\circ}$) and **c**, 2nd derivative of s-RC ($\text{d-RC} = \frac{d^2(\text{s-RC})}{dE^2}$) spectra. The dashed lines represent the A exciton (red), the interference background (gray) and absorption of the band edge (yellow).

Based on the anisotropic absorption of SiP_2 and the polarization-dependent RC spectra, we use the specific RC spectrum of the detection polarization along the y direction ($\pm 90°$) as the interference background to achieve the background-subtracted RC spectra ($\text{s-RC} = \text{RC} - \text{RC}_{\pm 90°}$). The s-RC and its 2nd derivative ($\text{d-RC} = \frac{d^2(\text{s-RC})}{dE^2}$)[31,32] are presented in Fig. S15b and S15c. Three peaks at 2.26 eV, 2.12 eV, and 2.05 eV can be observed in the d-RC spectrum. The first peak at 2.26 eV shows the absorption of band edges in bulk SiP_2. This value is consistent with the band edge absorption obtained from *GW*–RPA calculations (see Fig. S21). The peak at 2.12 eV originates from the F–P interference, as discussed above in Sec. 6-2. The last peak at 2.05 eV has exactly the same emission energy as the A exciton obtained in the PL measurement.

From the polarized absorption features, one can see that both the unconventional A exciton and the band edge in the RC spectra show the same linear dichroism character along the x direction of all measured samples (also see Fig. 3 in the Main Text and Fig. S10 in Sec. 4 of SI). This can be attributed to the anisotropic absorption of SiP_2. In other words, SiP_2 has a stronger absorption to the excitation light polarized along the x direction than that polarized along the y direction.

6-4 Comparison of experimental d-RC, calculated reflectance and absorbance

We compare the experimental reflectance spectrum with the calculated results from *GW*–BSE to determine more details of the exciton peaks. Herein, we simulate the absorptance and reflectance via calculating the dynamic dielectric for bulk SiP_2, in which eigen-energies of excitons are all accurately calculated. In the situation with low electron-hole density, the simulated PL spectra considered the photon-assisted process exactly correspond to discrete peaks for these eigenvalues of exciton states[33,34]. Figure S16 shows the measured d-RC spectrum, the calculated reflectance and absorbance spectra from *GW*–BSE calculations and their related derivatives. In these calculations, the ex–ph interactions are not considered. After a careful comparison of these features, we find that these peaks in absorbance correspond to the inflection points in reflectance,

which means that reflectance and absorbance spectra are consistent with each other in picking out the absorption features.

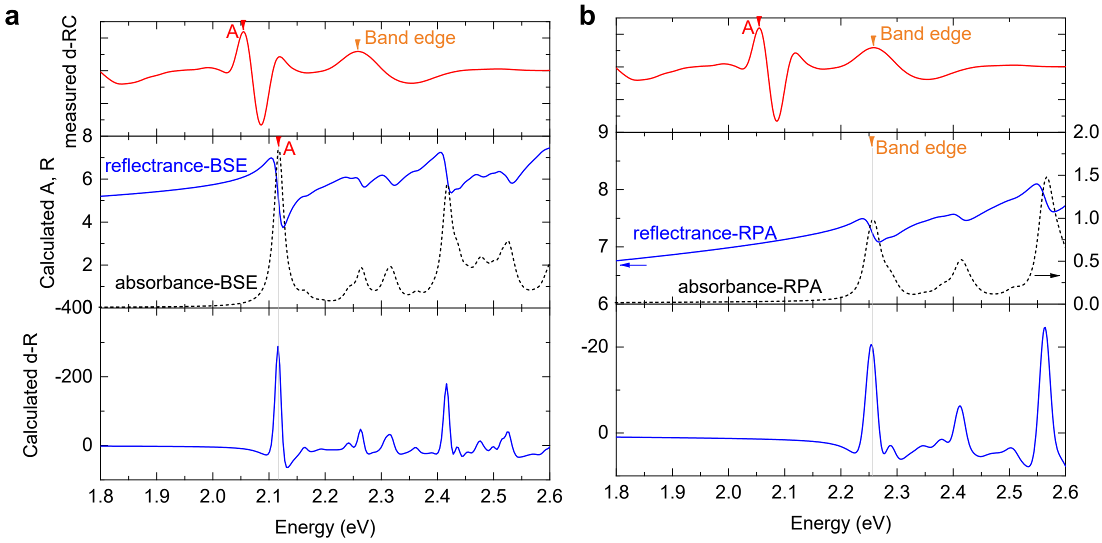

Figure S16 Comparison between the measured d-RC (red solid line) spectrum and calculated spectra of reflectance (blue solid line) and absorbance (black dashed line), as well as their derivatives. We present the calculated spectra based on **a**, *GW*–BSE and **b**, *GW*–RPA calculations for bulk SiP_2.

7. Temperature-dependent energy shifts of the band edge and A exciton

Figure S17 presents the temperature-dependent energy shifts of the optical band gap. One can see that the characteristic energy of the band edge shows a redshift as the temperature increases. The energy shift of the band edge is approximately 20 meV from 5.5 K to 300 K. To confirm the contribution from electron–phonon interactions to the optical band gap in bulk SiP_2, we performed a fitting procedure to the temperature-dependent energy shifts using the Bose–Einstein model[19,35–37]

$$E_g(T) = E_B - a_B\left(1 + \frac{2}{e^{E_0/k_B T} - 1}\right), \quad (2)$$

where E_g is the quasiparticle gap, E_B and a_B are fitting parameters, and E_0 is the effective phonon energy. Our fitting curve indicates a change in the band gap proportional to the effective Bose–Einstein distribution of phonons.

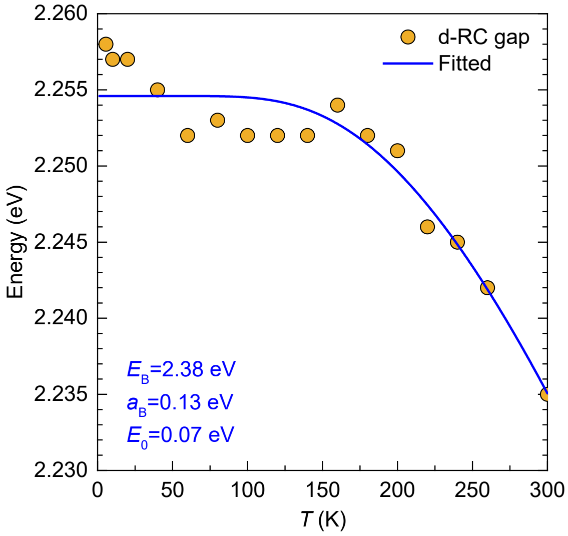

Figure S17 Temperature-dependent characteristic energies of the band edge extracted from the d-RC spectra. The solid blue line is the fitting result using the Bose–Einstein model. The fitting parameters are shown in the inset blue text.

Figure S18 presents temperature-dependent energy shifts of the unconventional A exciton, sideband A′, and their energy difference in different samples. As illustrated in Secs. 6 and 7, we can obtain the energy shift of the A exciton from the d-RC spectra (black circles filled by red). At the same time, we also obtained the energy shifts of the A exciton (red balls), sideband A′ (black balls), and the energy difference of A and A′ (violet balls) from the PL spectra.

Three important things need to be addressed here. First, for all the measured samples, the emission energies of the A exciton obtained from the d-RC spectra are perfectly consistent with those obtained from multipeak fitting of the PL spectra. This fact confirms the reproducibility of our measurements and the validation of the d-RC method in Sec. 6. Second, the redshifts of the A exciton are almost the same (~ 90 meV)

in PL/d-RC at temperatures from 5 K to 300 K. The redshifts of sideband peak A′ are slightly different because of the technical details in the fitting process and the broadening of the PL peak. The consistency of the large energy shifts of A and A′ among different samples confirms the reproducibility of the measurements as well as the robust and widely existing ex–ph coupling in SiP_2. Third, the energy differences of the A exciton and sideband A′ show similar behaviour in that the energy difference increases slightly from approximately 40 meV to 60 meV below 220 K. It drops sharply at temperatures higher than 220 K, which likely results from the fitting error at a higher temperature.

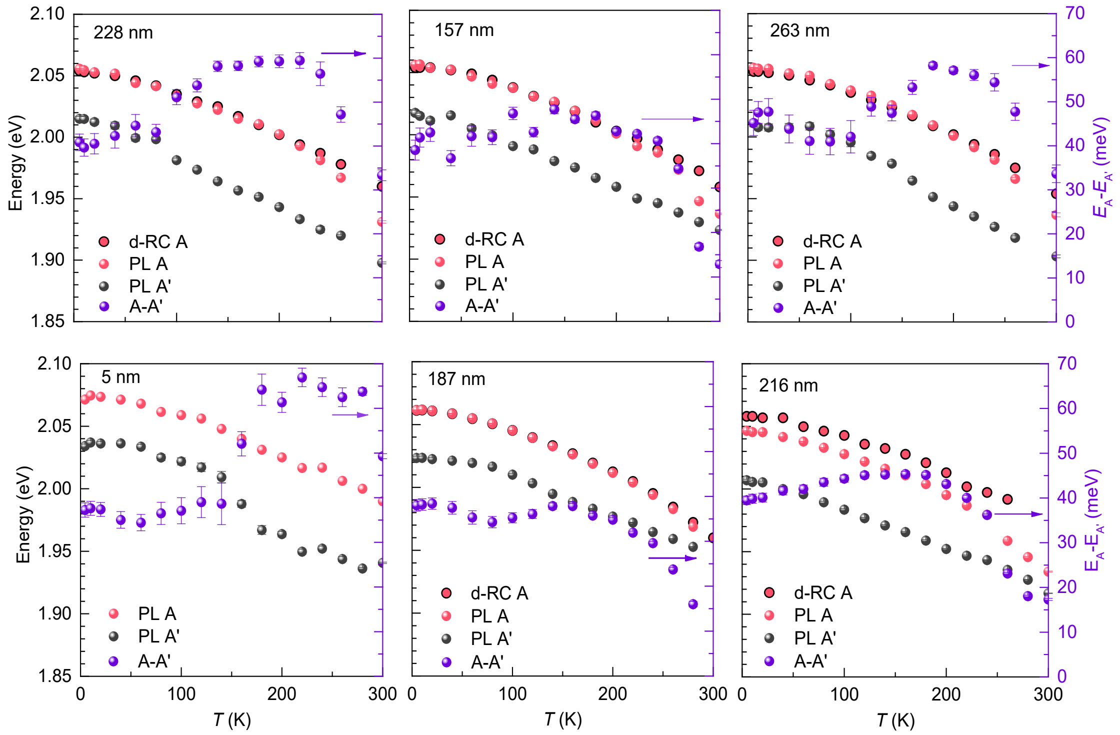

Figure S18 Temperature-dependent energies of the A exciton extracted by d-RC spectra (black circles filled by red), the A exciton (red sphere) fitted by PL spectra, and the sideband A′ (black sphere) fitted by PL spectra as well as the energy difference between A and A′ (violet sphere) obtained from six different samples with thicknesses from 5 nm to 263 nm.

8. Comparison of the phonon sideband and exciton–phonon coupling strength

As shown in Table S4, we summarized exciton energy (E_{exciton}), phonon sideband energy (E_{sideband}), energy difference $\Delta E = E_{\text{sideband}} - E_{\text{exciton}}$, the bandwidth, Huang-Rhys factor, and the intensity ratio between the zero-phonon line (ZPL) and the sideband feature ($I_{\text{sideband}}/I_{\text{ZPL}}$) of bulk SiP_2 and those materials previously reported with obvious exciton–phonon (ex–ph) interactions. Except for SiP_2, all the values in Table S4 for these materials are either given by or extracted from the references. For bulk SiP_2, the exciton binding energy and phonon sideband peak are calculated from *GW*–BSE and non-perturbative methods (see Methods section in the Main text). Huang-Rhys factors are estimated from the equation $S = \frac{M^2}{2E_{ph}^2}$, where M is the ex–ph coupling strength and E_{ph} is the corresponding phonon energy[38]. As shown in the Methods section in the Main Text, M is fitted to be 30 meV and E_{ph} is 55 meV. Consequently, the Huang-Rhys factor of SiP_2 is estimated to be 0.15, which is beyond the weak ex–ph coupling regime ($S \leq 0.1$)[38]. The relatively strong ex–ph coupling in SiP_2 can also be confirmed by its intensity ratio $I_{\text{sideband}}/I_{\text{ZPL}}$ (0.52), which is quite a large value compared to other materials.

Table S4. Comparison of the phonon sideband and exciton–phonon coupling strength

Material	Dimension	Method	$E_{exciton}$ (eV)	$E_{sideband}$ (eV)	ΔE (meV) $E_{sideband} - E_{exciton}$	E_{ph} (meV)	Bandwidth (eV)			Huang-Rhys factor	$I_{sideband}/I_{ZPL}$	Reference
							direction	e	h			
SiP_2	3D	PL, Reflectance, Absorption	2.06	2.01	−50		X–S<br>X–Γ–Z	1.63<br>0.08	1.37<br>0.64	0.15	0.52	This work
(6,5) CNT	1D	PL, PLE, Absorption	1.258	1.124<br>1.464	−134<br>206	326					0.05<br>0.11	[39]
(6,5) CNT	1D	Absorption, Pump-probe								0.03 (RBM)<br>0.4 (LO)		[40]
(10,2), (9,4), (8,6), (12,1), (10,5) CNTs	1D	PL, Raman								0.013 ~ 0.025		[38]
1L-Tetracene	2D	PL, Reflectance	2.35	2.44	90	93					0.58	[41],[42]
$In_xGa_{1-x}N/GaN$ Multi-QWs	2D	PL	3.268	3.181 (1LO)<br>3.102 (2LO)<br>3.019 (3LO)<br>2.931 (4LO)	−87<br>−79<br>−83<br>−88	86					0.69<br>0.35<br>0.17<br>0.11	[43]
$GaN/Al_xGa_{1-x}N$ Multi-QWs	2D	PL	3.692	3.625 (1TO)<br>3.558 (2TO)<br>3.489 (3TO)	−67<br>−67<br>−69	68					0.54<br>1.30<br>0.31	[43],[44]
ZnSe/ZnSSe Multi-QWs	2D	PL	2.815	2.784 (1LO)	−31	32					0.01	[45],[46]
GaN	3D	PL	3.495	3.410 (1LO)<br>3.315 (2LO)	−85<br>−95	91	Γ–K<br>Γ–M	4.7<br>2.8	2.8<br>1.2		0.04<br>0.01	[47],[48]
ZnO	3D	PL	3.376	3.310<br>3.237<br>3.166<br>3.093	−66<br>−63<br>−71<br>−73	74	Γ–K<br>Γ–M	6.6<br>4.8	2.2<br>0.9		0.77<br>0.49<br>0.24<br>0.15	[49],[50]

*QW: quantum well. PLE: PL excitation spectrum. LO: longitudinal optical phonon. TO: transverse optical phonons. RBM: radial breathing mode.

9. Pump-probe measurements and linearly polarized transient reflection

In order to understand the depolarization dynamics of photoexcited excitons, we performed pump-probe transient optical measurements. Pump-probe measurements were based on a Yb-base amplifier (Pharos, Light Conversion), providing high-power diode lasers in the λ_{pump} = 480 ~ 680 nm wavelength range for pumping. The temporal resolution is better than 10 fs when using a pump beam of the home-built noncollinear optical parametric amplifier and a probe beam of a supercontinuum source covering 600 ~ 900 nm. The pumping laser was focused on a spot with a diameter of ~ 10 μm and had a pump density of ~ 200 μJ/cm^2. As shown in the pump-probe reflection measurement geometry in Fig. S19a, the polarization of the pump pulse is fixed along the x-axis of SiP_2 (red arrow), while the polarization of the probe pulse is set to either the x-axis (across the P_B–P_B chain) or y-axis direction (along the P_B–P_B chain) of SiP_2. The differential reflection signal $\Delta R/R$ was collected, where R represents the reflection signal from the sample without the pump pulse and ΔR represents the difference in the reflection signal from the sample with and without the pump pulse.

Figure S19b shows the pump-probe results at a probe wavelength of 640 nm (corresponding to an A exciton of SiP_2 at room temperature) along the x- and y-axes. Under laser pumping, we expect that only this A excitonic state can be excited without other relaxation processes being involved and the change in the photoinduced reflectivity shows a strong dependence on the probe polarization. An ultrafast process with a lifetime τ_u ~ 250 fs is only observed within the probe polarization along the x direction (the polarization direction of the exciton in SiP_2), which is probably related to the depolarization dynamics of the anisotropic bound excitonic state. After this ultrafast process, we can observe similar slow relaxation processes both for the probe polarization along the x- and y-axes with relaxation times ($\tau_x = \tau_y$ ~ 3.2 ps).

To conclude, based on our pump-probe ultrafast optical measurement, the lifetime of the unconventional exciton in SiP_2 (dissolved to unbound electrons and holes) is ~ 250

fs, corresponding to a linewidth broadening of ~ 20 meV (roughly estimated from the uncertainty principle). This value obtained from ultrafast measurement is roughly consistent with the linewidth in our PL measurement (Fig. S12). We argue that the ex–ph[17,51] interactions in bulk SiP_2 could be the main reason to dissolve the unconventional exciton in SiP_2.

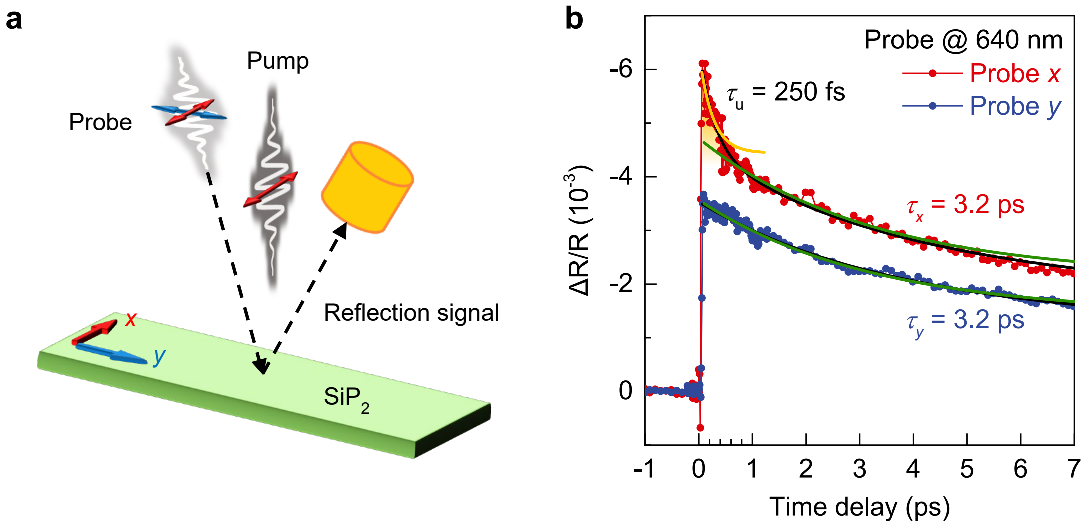

Figure S19 **a**, Schematic diagram of the pump-probe measurement geometry. **b**, Time-resolved reflectance results with a probe wavelength equal to 640 nm and probe pulse polarization along the x-axis (red dots) and y-axis (blue dots). The solid black lines are the corresponding fitting results to multiexponential functions. An ultrafast process (solid orange line) with a lifetime τ_u of ~ 250 fs along the x direction (across the P_B–P_B chain) is only captured from the signal (red dots), while there is no such relaxation process with the polarization of the probe laser along the y direction along the P_B–P_B chain (blue dots). Another relaxation process (solid green lines) with a lifetime $\tau_x = \tau_y \sim 3.2$ ps can be observed in both measurement geometries.

10. Lattice structures from DFT calculations

We use different exchange-correlation functionals to fully relax the lattice structure of bulk SiP_2 with the space group No. 62 (*Pnma*) and obtain the lattice constants shown

in Table S5. The lattice constants, including the GGA–PBE functional with the vdWs correction, are closer to the experimental values from STEM than those obtained using GGA–PBE and LDA functionals. This indicates that the vdWs corrections are important to determine the structural and electronic properties of bulk SiP_2.

Table S5. The lattice constants of bulk SiP_2 (Space Group No. 62)

	a **(Å)**	***b*** **(Å)**	***c*** **(Å)**
GGA–PBE	10.301	3.448	15.207
LDA	9.810	3.404	13.725
vdWs corrections	10.156	3.465	14.146
Our TEM result	10.1	3.4	14.0

11. Calculated electronic band structures of bulk SiP_2 based on different exchange-correlation functionals

The calculated band structures of bulk SiP_2 are shown in Fig. S20, whose lattice structure is fully relaxed with vdWs correction. The calculation details can be found in the Methods section of the Main Text. The GGA–PBE functional with vdWs correction strongly underestimates the indirect band gap of bulk SiP_2 by approximately 0.8 eV. Furthermore, we found that the calculated valence band maximum (VBM) is at the Γ point and the conduction band minimum (CBM) is at the X point. This result is in contrast to that from *GW* calculations (see Fig. 1e in the Main Text). At the same time, we found that the electronic structures of bulk SiP_2 from conventional DFT calculations depend on the choice of exchange-correlation functionals. However, the observations of embedded quasi-1D electron states and quasi-2D hole states are robust.

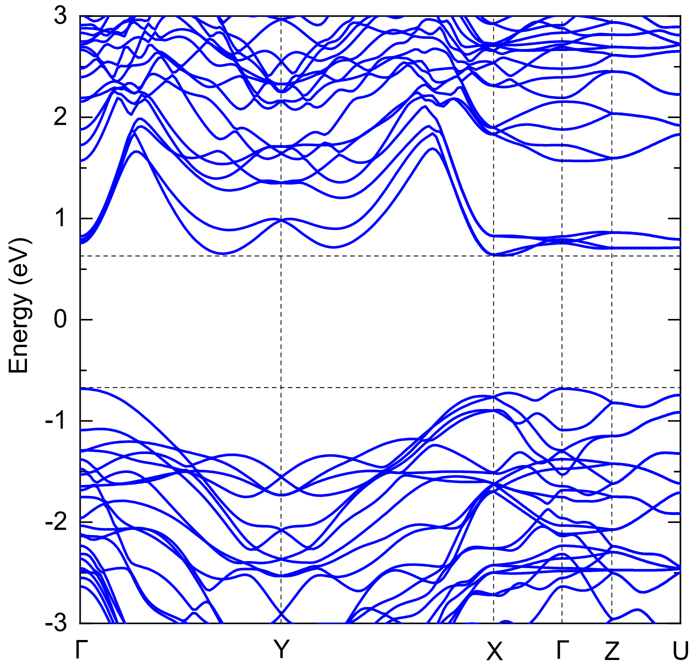

Figure S20 Calculated electronic band structure of bulk SiP_2 with the GGA–PBE exchange-correlation functional and vdWs corrections. We fully relax the lattice structures with the vdWs correction to obtain the lattice constants. The middle of the band gap is set as the Fermi level at zero energy. The calculated band gap is 1.28 eV, which is much smaller than that from the *GW* calculation, which is 2.14 eV.

12. Excitons from *GW*–BSE calculations for bulk SiP_2

12-1 Absorption from *GW*–BSE calculations for bulk SiP_2

Figure S21 reports the simulated absorption spectra of bulk SiP_2 along different directions, which are the imaginary part of the calculated dielectric response function obtained from the *GW* calculation within the random phase approximation (RPA) and by solving the *GW*–BSE. The calculated binding energy of such an unconventional exciton is approximately 140 meV, which is defined as the energy difference between the exciton peak and conduction band edge at the X point. Consistent with the experimental PL and RC observations, there are only absorption signals along the *x* and *z* directions for exciton peak A and the band edge due to the crystal symmetry constraints in bulk SiP_2. Since in the PL and RC experiments, the propagation directions

of the incident and reflected light are along the *z* direction of bulk SiP_2 and the electric field components of the light oscillate in the *xy* plane, we cannot observe absorption along the *z* direction in our experiments.

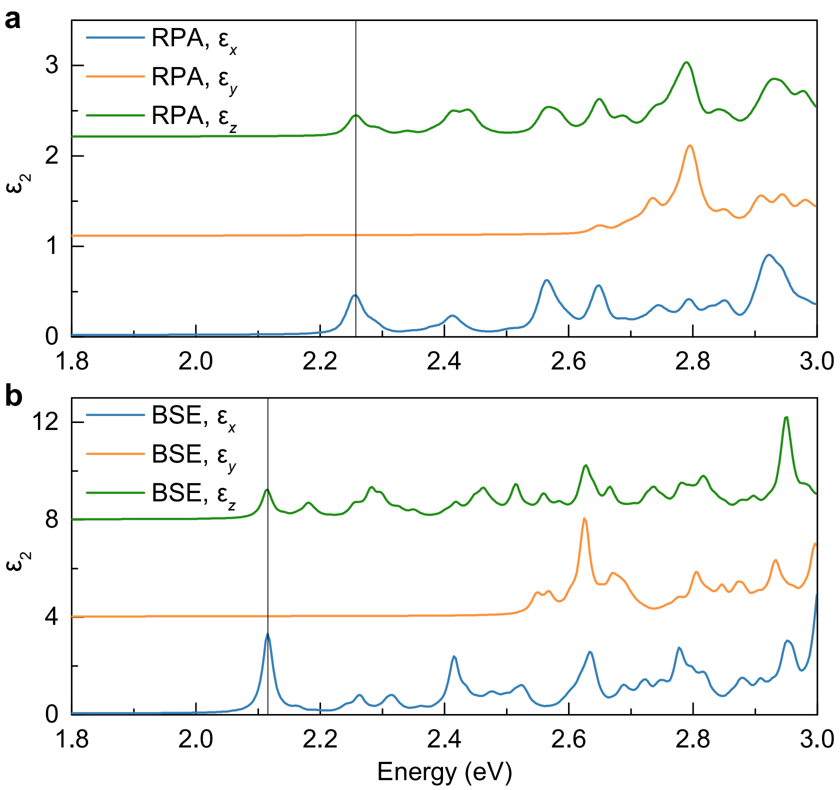

Figure S21 The simulated absorption described by the imaginary part of the dielectric constant of bulk SiP_2 calculated from **a**, *GW*–RPA and **b**, *GW*–BSE along the *x* (dark blue), *y* (orange) and *z* (green) directions. The black dashed line depicts the direct *GW* band gap at the X point.

12-2 Decomposition of the A exciton wavefunction into electronic states in k space

The wavefunction of the excitonic state $\psi_\mu(\mathbf{r}_h, \mathbf{r}_e)$ can be written as

$$\psi_\mu(\mathbf{r}_h, \mathbf{r}_e) = \sum_{v,c,\mathbf{k}} A^{\mu}_{v,c,\mathbf{k}} |e_{c,\mathbf{k}}(\mathbf{r}_e)\rangle \otimes |h_{v,\mathbf{k}}(\mathbf{r}_h)\rangle$$

where $\mathbf{r}_h$ is the position of the hole, $\mathbf{r}_e$ is the position of the electron, v is the valence band index, c is the conduction band index, and $\mathbf{k}$ is the momentum. The functions $e_{c,\mathbf{k}}(\mathbf{r}_e)$ and $h_{v,\mathbf{k}}(\mathbf{r}_h)$ represent the quasiparticle states of electrons and holes. In reciprocal space, the exciton μ can be visualized by computing the weights of the conduction and valence bands via

$$W^{\mu}_{c,\mathbf{k}} = \sum_{v} |A^{\mu}_{v,c,\mathbf{k}}|^2$$

and

$$W^{\mu}_{v,\mathbf{k}} = \sum_{c} |A^{\mu}_{v,c,\mathbf{k}}|^2$$

We plotted the distribution of the weights of the conduction and valence bands for the A exciton in bulk SiP_2 in reciprocal space, as shown in Fig. S22. The A excitons are mainly contributed by electrons confined along one dimension and holes confined in the X–Γ–Z plane of the first BZ (e.g., from the band edge states at the X point). Therefore, we conclude that the observed A exciton from the PL and RC experiments is an unconventional excitonic state. Fig. S22 also shows the bandwidths for the conduction band edge and valence band edge calculated from the *GW* calculation. The bandwidths of the conduction band and valence band in the X–Γ–Z plane of the first BZ are approximately 0.08 eV and 0.64 eV, respectively, which are an order of magnitude smaller than those along the X–S line of the first BZ.

In the discussion of the Main Text and in Sec. 13 of the SI, we treat the X point as the representative **k**-point, and then we calculate its electronic state to explore the influence of electron–phonon interactions (see Fig. 2 in the Main Text) and to discuss crystal symmetry constraints (see Sec. 12-3).

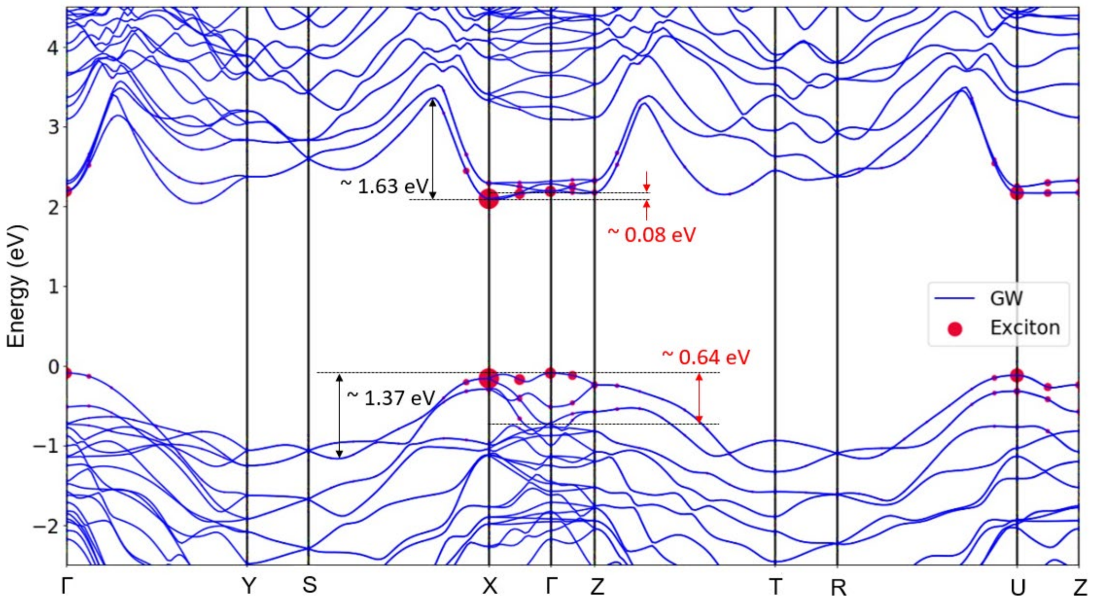

Figure S22 Reciprocal space distribution of the excitonic wavefunction of the A exciton after solving *GW*–BSE. The band structures are obtained from *GW* calculations. The energy of the VBM is set as the Fermi level at zero energy. The size of the red dots is proportional to the excitonic weights *W* of the conduction and valence bands for the A exciton. The black and red lines with arrows represent the bandwidth for the conduction band edge and valence band along the X–S line and in the X–Γ–Z plane of the first BZ. The black dotted lines mark the energy positions on which we count for bandwidths.

12-3 Symmetry analysis of the electronic structures of bulk SiP_2 at the X point

Bulk SiP_2 hosts space group No. 62 containing eight group elements, in which there are three basic group generators: inversion symmetry *P*, nonsymmorphic screw symmetries along the *x*-axis $S_{2x} = \{C_{2x}|\frac{1}{2},\frac{1}{2},\frac{1}{2}\}$, and screw symmetry along the *y*-axis $S_{2y} = \{C_{2y}|0,\frac{1}{2},0\}$. No magnetism is observed in bulk SiP_2; thus, the time-reversal symmetry *T* is not broken. Its lattice constants are labeled *a*, *b*, and *c,* and their values are shown in Table S5.

From our *GW* calculations, the conduction band edge at the X point in the first BZ is very close to the CBM of bulk SiP_2. Although the VBM is at the Γ point, the valence

band edge at the X point is also very close to the VBM. Based on our *GW*–BSE calculations, electrons and holes at the X point play an important role in forming an A exciton (see Sec. 12-2 for details). Therefore, in the following, we focus on the electronic states around the X point. The Bloch states at the X point are eigenstates of P, S_{2y}, and S_{2x} symmetries.

Due to the P and T symmetries in bulk SiP_2, electronic states at arbitrary **k** points are doubly degenerate. Because of the small spin–orbit coupling in bulk SiP_2, we treated bulk SiP_2 as a spinless system. μ_h and μ_c are the eigenvalues of inversion symmetry for the valence band edge and conduction band edge at the X point. For the screw symmetry along the *x*-axis, we have

$$(S_{2x})^2 = T(1,1,1) = e^{i(k_x+k_y+k_z)}$$

For the Bloch state as an eigenstate of S_{2x}, its eigenvalue is $g_{\pm}^{x} = \lambda^x e^{i(k_x+k_y+k_z)/2}$, where $\lambda^x = \pm 1$ labels two distinct representations. For the state at the X point, we have $g_{\pm}^{x} = i\lambda^x$.

For the screw symmetry along the *y*-axis, we have

$$(S_{2y})^2 = T(0,1,0) = e^{ik_y}$$

For the Bloch state as an eigenstate of S_{2y}, its eigenvalue is $g_{\pm}^{y} = \lambda^y e^{ik_y/2}$, where $\lambda^y = \pm 1$ labels two distinct representations. For the state at the X point, we have $g_{\pm}^{y} = \lambda^y$.

The PL spectra are determined by the absorption matrix elements that are constrained by crystal symmetry. For example, the absorption along the *x*(*y*) direction is determined by matrix element $|\langle c_X|x(y)|h_X\rangle|^2$, where $|h_X\rangle$ and $|c_X\rangle$ are the Bloch states at the valence band edge and conduction band edge at the X point. Under symmetries of P, S_{2y}, and S_{2x}, we have

$$P: (x, y, z) \to (-x, -y, -z)$$

$$P|h_X\rangle = \mu_h|h_X\rangle$$

$$P|c_X\rangle = \mu_c|c_X\rangle$$

$$S_{2y}: (x, y, z) \to (-x, y + \frac{b}{2}, -z)$$

$$S_{2y}|h_X\rangle = \lambda_h^y|h_X\rangle$$

$$S_{2y}|c_X\rangle = \lambda_c^y|c_X\rangle$$

$$S_{2x}: (x, y, z) \to (x + \frac{a}{2}, -y + \frac{b}{2}, -z + \frac{c}{2})$$

$$S_{2x}|h_X\rangle = i\lambda_h^x|h_X\rangle$$

$$S_{2x}|c_X\rangle = i\lambda_c^x|c_X\rangle$$

For the matrix element $\langle c_X|x(y)|h_X\rangle$, we have

$$\langle c_X|x(y)|h_X\rangle = \langle c_X|P^{-1}Px(y)P^{-1}P|h_X\rangle = -(\mu_c)^*\mu_h\langle c_X|x(y)|h_X\rangle$$

$$\langle c_X|x|h_X\rangle = \langle c_X|(S_{2y})^{-1}S_{2y}x(S_{2y})^{-1}S_{2y}|h_X\rangle = -(\lambda_c^y)^*\lambda_h^y\langle c_X|x|h_X\rangle$$

$$\langle c_X|y|h_X\rangle = \langle c_X|(S_{2y})^{-1}S_{2y}y(S_{2y})^{-1}S_{2y}|h_X\rangle = (\lambda_c^y)^*\lambda_h^y\langle c_X|y|h_X\rangle$$

$$\langle c_X|x|h_X\rangle = \langle c_X|(S_{2x})^{-1}S_{2x}x(S_{2x})^{-1}S_{2x}|h_X\rangle = (\lambda_c^x)^*\lambda_h^x\langle c_X|x|h_X\rangle$$

$$\langle c_X|y|h_X\rangle = \langle c_X|(S_{2x})^{-1}S_{2x}y(S_{2x})^{-1}S_{2x}|h_X\rangle = -(\lambda_c^x)^*\lambda_h^x\langle c_X|y|h_X\rangle$$

Therefore, if $(\mu_c)^*\mu_h = 1$, we have $\langle c_X|x(y)|h_X\rangle = 0$. If $(\lambda_c^y)^*\lambda_h^y = 1$, $\langle c_X|x|h_X\rangle = 0$, while $\langle c_X|y|h_X\rangle \neq 0$; and $(\lambda_c^y)^*\lambda_h^y = -1$, $\langle c_X|x|h_X\rangle \neq 0$ while $\langle c_X|y|h_X\rangle = 0$. If $(\lambda_c^x)^*\lambda_h^x = 1$, $\langle c_X|x|h_X\rangle \neq 0$ while $\langle c_X|y|h_X\rangle = 0$; and $(\lambda_c^x)^*\lambda_h^x = -1$, $\langle c_X|x|h_X\rangle = 0$, while $\langle c_X|y|h_X\rangle \neq 0$.

In our DFT calculation, by using the GGA–PBE function with vdWs corrections, we calculated eigenvalues of these crystal symmetries for the conduction band edge and valence band edge at the X point for bulk SiP_2. Due to the double degeneracy, we used ↑ and ↓ to label these degenerated states. For the inversion symmetry *P*, we found $\mu_h^{\uparrow} = -1$, $\mu_h^{\downarrow} = 1$, $\mu_c^{\uparrow} = -1$, and $\mu_c^{\downarrow} = -1$; therefore, $\left\langle c_X^{\uparrow(\downarrow)} \middle| x(y) \middle| h_X^{\uparrow} \right\rangle = 0$. For the screw symmetry S_{2y}, we found $\lambda_h^{y\uparrow} = -1$, $\lambda_h^{y\downarrow} = 1$, $\lambda_c^{y\uparrow} = -1$, and $\lambda_c^{y\downarrow} = -1$. We have $\left\langle c_X^{\uparrow(\downarrow)} \middle| y \middle| h_X^{\downarrow} \right\rangle = 0$ and $\left\langle c_X^{\uparrow(\downarrow)} \middle| x \middle| h_X^{\downarrow} \right\rangle \neq 0$. Therefore, guaranteed by the crystal symmetries of bulk SiP_2, only absorption along the *x* direction can be observed. These discussions are consistent with the calculated results obtained from the *GW*–RPA and *GW*–BSE calculations shown in Fig. S21 and the experimental observations shown in Fig. 3 in the Main Text. Furthermore, we plotted the modulus squared of the A exciton's wavefunction in real space distribution in Fig. 3c of the Main Text.

13. Influence on electronic bands by electron–phonon interactions for bulk SiP_2

13-1 Calculated phonon band structures and optical phonon modes for bulk SiP_2

In our *ab initio* calculation of the phonon band structure for bulk SiP_2, we consider the influence of the polar longitudinal-optical (LO) phonons by adding the nonanalytic contribution (NAC) to the dynamical matrix, in which the dipole–dipole interactions induced by the polar phonon vibrations are considered. This method has been widely used for the *ab initio* calculation of phonons for polar semiconductors and insulators[52–54]. The dynamic matrix $D_{\alpha\beta}$ has the form

$$D_{\alpha\beta}(jj', \boldsymbol{q} \to 0) = D_{\alpha\beta}(jj', \boldsymbol{q} = 0) + \frac{1}{\sqrt{m_j m_{j'}}} \frac{4\pi}{\Omega_0} \frac{[\sum_{\gamma} q_{\gamma} Z^{*}_{j,\gamma\alpha}][\sum_{\gamma'} q_{\gamma'} Z^{*}_{j',\gamma'\beta}]}{\sum_{\alpha\beta} q_{\alpha} \varepsilon^{\infty}_{\alpha\beta} q_{\beta}}$$

where $D_{\alpha\beta}(jj', \boldsymbol{q} = 0)$ is the dynamical matrix of phonons calculated from DFT within the frozen-phonon approach. The second term includes the effect of the polar

LO phonon, $m_{j(j')}$ is the atomic mass for the $j(j')$-th atom in SiP_2, $\boldsymbol{q}$ is the wavevector of the phonon mode, Ω_0 is the volume of the unit cell, $Z^*_{j,\gamma\alpha}$ is the effective Born charge for the jth atom, and $\varepsilon^{\infty}_{\alpha\beta}$ is the dielectric constant. We calculated $\varepsilon^{\infty}_{\alpha\beta}$ and $Z^*_{j,\gamma\alpha}$ by using density functional perturbation theory, whose values are shown in Table S6. Because of the anisotropic lattice structure of bulk SiP_2, the dielectric constant and effective Born charge are also anisotropic.

Table S6. The dielectric constant (DC) $\boldsymbol{\varepsilon^{\infty}_{\alpha\beta}}$ and the effective Born charge (EBC) $\boldsymbol{Z^*_{j,\gamma\alpha}}$ for bulk SiP_2.

DC	ε_{xx}	ε_{xy}	ε_{xz}	ε_{yx}	ε_{yy}	ε_{yz}	ε_{zx}	ε_{zy}	ε_{zz}
	9.60	0.00	0.00	0.00	10.94	0.00	0.00	0.00	7.75
EBC	Z^*_{xx}	Z^*_{xy}	Z^*_{xz}	Z^*_{yx}	Z^*_{yy}	Z^*_{yz}	Z^*_{zx}	Z^*_{zy}	Z^*_{zz}
Si-1	1.87	0.00	−0.88	0.00	1.92	0.00	−0.22	0.00	0.52
Si-5	1.67	0.00	−1.19	0.00	1.77	0.00	−0.59	0.00	1.03
P-1	−1.21	0.00	0.75	0.00	−1.62	0.00	0.38	0.00	−0.70
P-5	−0.56	0.00	0.38	0.00	−0.14	0.00	0.07	0.00	0.18
P-9	−0.12	0.00	−0.02	0.00	−0.26	0.00	0.03	0.00	−0.76
P-13	−1.67	0.00	0.19	0.00	−1.67	0.00	0.30	0.00	−0.38

* The atomic index corresponds to Figure S3. The unit of effective Born charge is the charge of the electron. Due to the crystal symmetry, two Si atoms and four P atoms exist in SiP_2 independently.

We plot the phonon band structures of bulk SiP_2 with and without considering the NAC correction in Fig. S23a. We can observe longitudinal optical-transverse optical (LO–TO) splitting at the Γ point for some optical phonon modes when their wave vectors **q** are along the Γ–X and Γ–Y directions. However, the value of LO–TO splitting is relatively small compared with some traditional polar oxides, e.g., $Z_{Ti}^* = 7.4$ in $SrTiO_3$[55–57].

There is no negative frequency in the whole first BZ, which suggests that bulk SiP_2 within the space group of *Pnma* is stable. In one unit cell of bulk SiP_2, 24 atoms contribute to 72 phonon modes. In Fig. S23b, we show a zoomed-in view of the optical phonon bands in the energy range from 45 meV to 67 meV.

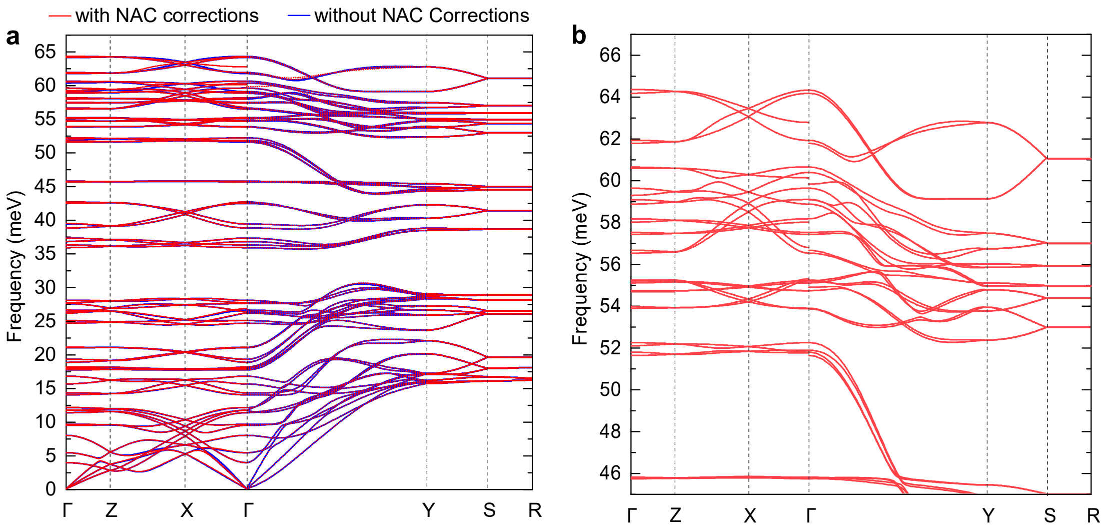

Figure S23 **a**, The phonon band structure for bulk SiP_2 with (red line) and without (blue line) considering the NAC corrections. For red lines, LO–TO splitting can be observed clearly. We fully relax the lattice structures by using the GGA–PBE functional with vdWs corrections to obtain the lattice constant. **b**, Zoomed-in plot for optical phonon modes in the energy range from 45 meV to 67 meV.

13-2 Band gap change from electron–phonon interaction within a "frozen-phonon" scheme

Based on the quantum theory of phonons, the atomic displacement $\mathbf{u}_{\alpha}(jl,t)$ of the phonon mode along the α direction of the jth atom in the lth unit cell has the form of

$$\mathbf{u}_\alpha(jl,t) = (\frac{\hbar}{2Nm_j})^{1/2} \sum_{\mathbf{q},\nu} \omega_\nu(\mathbf{q})^{-1/2} \, [b_\nu(\mathbf{q}) e^{-i\omega_\nu(\mathbf{q})t}$$

$$+ b_\nu^\dagger(-\mathbf{q}) e^{i\omega_\nu(\mathbf{q})t}] e^{i\mathbf{q}\cdot\mathbf{r}(jl)} \mathbf{e}_{\alpha,\mathrm{j},\nu}(\mathbf{q})$$

where $b_\nu^\dagger(-\mathbf{q})$ and $b_\nu(\mathbf{q})$ are the phonon creation and annihilation operators at the $-\mathbf{q}$ and $\mathbf{q}$ points, $\hbar$ is the reduced Planck constant, N is the number of atoms in one unit cell (N = 24 for bulk SiP_2), m_j is the mass of the jth atom, $\mathbf{e}_{\alpha,j,\nu}(\mathbf{q})$ is the eigenvector of the phonon mode with band index ν at the $\mathbf{q}$ point, and $\omega_\nu(\mathbf{q})$ is the corresponding phonon frequency. Therefore, the expectation value of the mean squared displacement for each phonon mode is

$$\langle [u_{\alpha,j}]^2 \rangle = \frac{\hbar}{2Nm_j} \sum_{\mathbf{q},\nu} \omega_\nu(\mathbf{q})^{-1} \left[\mathbf{e}_{\alpha,j,\nu}(\mathbf{q})\right]^2 (1 + 2n_\nu(\mathbf{q}))$$

in which $n_\nu(\mathbf{q})$ is the phonon population given by the Bose–Einstein distribution

$$n_\nu(\mathbf{q}) = \frac{1}{e^{\frac{\hbar\omega_\nu(\mathbf{q})}{k_\mathrm{B}T}} - 1}$$

T is the temperature, and k_B is the Boltzmann constant. In the framework of the frozen-phonon approximation, we could use the mean-squared displacement to estimate the displacement for each phonon mode[58].

Following the previous discussion[58], the shift of an electronic eigenenergy $\Delta E_{n,\mathbf{k}}$ of band n and wave vector **k** has the form of

$$\Delta E_{n,\mathbf{k}} = \sum_\nu \sum_\mathbf{q} \frac{\partial E_{n,\mathbf{k}}}{\partial n_\nu(\mathbf{q})} \left(n_\nu(\mathbf{q}) + \frac{1}{2} \right)$$

and $\frac{\partial E_{n,\mathbf{k}}}{\partial n_\nu}$ can be calculated directly in the framework of density functional perturbation theory or the frozen phonon approximations[59].

Here, we used frozen phonon approximations to estimate the change in electronic

structures induced by electron–phonon interactions and resolved this influence for each optical phonon mode. We estimated the change in electronic bands by averaging the obtained energy shifts with atomic displacement $\pm\boldsymbol{\chi}_{\nu}(\mathbf{q})$, which was obtained from phonon calculations. This has the form of

$$\boldsymbol{\chi}_{\nu}(\mathbf{q}) = (\frac{\hbar}{Nm_j})^{1/2}\omega_{\nu}(\mathbf{q})^{-1/2}\mathbf{e}_{\alpha,j,\nu}(\mathbf{q})$$

Because the unconventional exciton is mainly contributed by the electron and hole at the X point, we approximate the band shifts induced by electron–phonon interaction using the phonon mode at the Γ point $(\mathbf{q} = 0)$. As shown in Fig. S24 and Fig. 2e in the Main Text, we plot the band gap shifts E_g^{X} at the X point for each optical phonon at the Γ point.

By using the PBE exchange-correlation functional with the vdWs corrections, we first calculated the atomic displacement $\boldsymbol{\chi}_{\nu}(\mathbf{q} = 0)$ for each optical phonon mode; then, we performed the DFT calculation with the lattice structure containing atomic displacements $\pm\boldsymbol{\chi}_{\nu}(\mathbf{q} = 0)$ and obtained the band gap shifts at the X point induced by each optical phonon displacement in the framework of the frozen phonon approach. The band gap shift E_g^{X} is defined as $E_g^{\mathrm{X}} = E_g^{\mathrm{X}}|_{L_0\pm\chi_\nu} - E_g^{\mathrm{X}}|_{L_0}$, where $E_g^{\mathrm{X}}|_{L_0}$ and $E_g^{\mathrm{X}}|_{L_0\pm\chi_\nu}$ are the band gaps at the X point without and with displacement $\pm\boldsymbol{\chi}_{\nu}(\mathbf{q} = 0)$ of the phonon modes. We found that phonon modes #35, #47, #49, #50, #65, #66, #67, and #68 (as shown in Fig. S24) mainly contribute to a larger E_g^{X} in comparison to that from other phonon modes. The numbers that label the phonon mode denote the indices of the phonon modes sorted in order of increasing frequency.

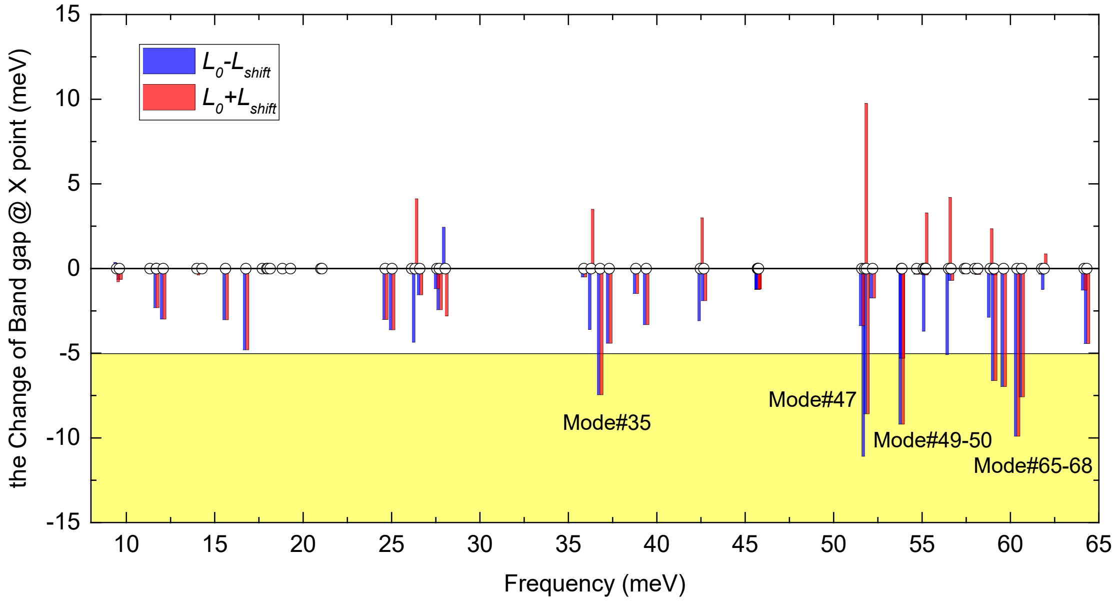

Figure S24 The band gap shifts at the X point induced by each optical phonon mode at the Γ point, which are calculated in the framework of the frozen phonon approach. Electronic structures and phonon modes are calculated by using the exchange-correlation functional with vdWs corrections. L_0 represents the lattice structure without displacement of phonon modes. $\pm L_{\text{shift}} = \pm \boldsymbol{\chi}_{\nu,\mathbf{q}=0}$ is the displacement of phonon modes. Black circles represent the energies of corresponding phonon modes at the Γ point along the Γ–Y direction. Herein, we mark the indices of some phonon modes that have relatively larger contributions to electron–phonon interactions.

Their lattice vibrations in real space are shown in Fig. S25. These vibration modes mainly involve the embedded P_B–P_B chains along the *xz* plane or adjacent Si atoms, which strongly modify the electronic potential of the embedded states with hybrid dimensionality in bulk SiP_2, resulting in relatively strong electron–phonon coupling. Although there is a large phonon density of states in the energy range from 43 meV to 50 meV (see Fig. 2g in the Main Text), which is mainly contributed by embedded P_B–P_B chains, the vibrations of these modes are along the *y* direction and do not couple with the electronic states in the X–Γ–Z plane of the first BZ.

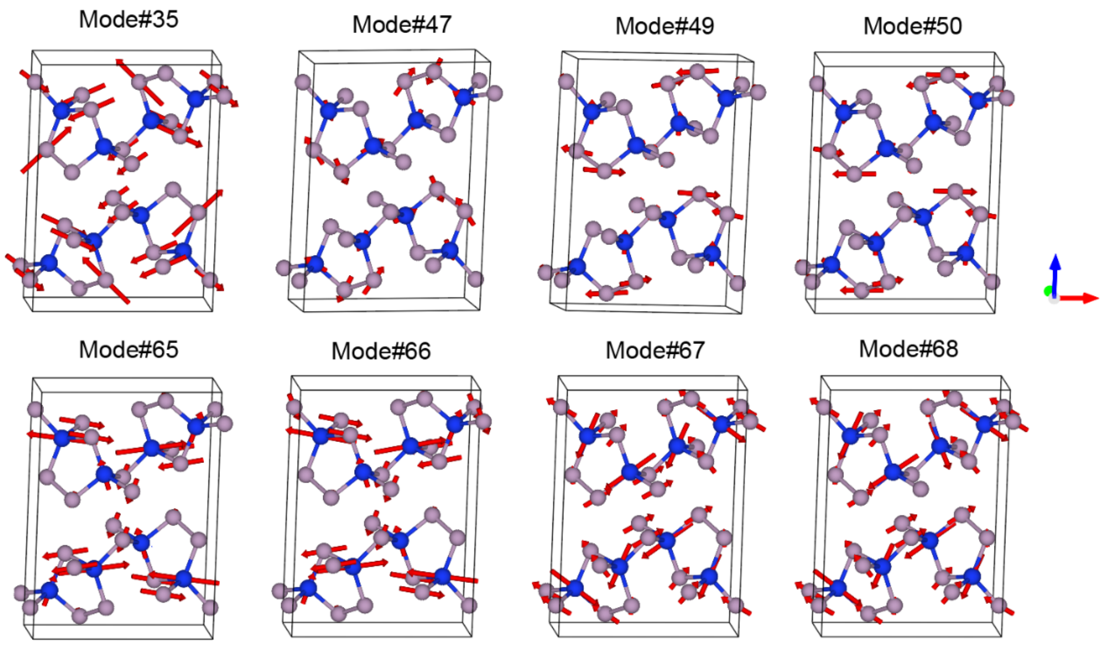

Figure S25 Lattice vibrations of phonon modes #35, #47, #49, #50, #65, #66, #67, and #68 in real space. The vibrations of phonon modes mainly couple with the P_B–P_B chains and the adjacent Si atoms in bulk SiP_2. All the vibrations of these atoms are in the *xz* plane.

13-3 Justification of non-perturbation theory for the description of exciton–phonon interactions in bulk SiP_2

As discussed above (Sec. 12 in SI), the wavefunction of the exciton can be expressed as $\psi_\mu(\mathbf{r}_h, \mathbf{r}_e) = \sum_{v,c,\mathbf{k}} A^{\mu}_{v,c,\mathbf{k}} |e_{c,\mathbf{k}}(\mathbf{r}_e)\rangle \otimes |h_{v,\mathbf{k}}(\mathbf{r}_h)\rangle$, as defined in Sec. 12. Thus, the larger the electron–phonon and hole–phonon interactions are, the larger the ex–ph coupling is[60]. Considering the contribution from polar phonons, we argue that the dimensionless Fröhlich polaron constant $\alpha_{e/h}$ is a good quantity to depict these interactions between electrons (holes) and optical phonons qualitatively[61], and the Fröhlich polaron constant is

$$\alpha_{e/h} = \frac{e^2}{\hbar}\left(\frac{m_{e/h}}{2\hbar\omega_{LO}}\right)^{\frac{1}{2}}\left(\frac{1}{\varepsilon_\infty} - \frac{1}{\varepsilon_0}\right)$$

where e is the electron charge, $\hbar$ is the reduced Planck constant, ω_{LO} is the phonon frequency for the longitudinal optical phonon modes, the dielectric constant ε_∞ is the electronic component of the dielectric screening, ε_0 is the low-frequency dielectric constant, and $m_{e/h}$ is the effective mass for electron and hole states.

In bulk SiP_2, the absorption of the A exciton only has a component along the *x* direction across atomic P_B–P_B chains (see Fig. 3 in the Main Text). The corresponding electron states have relatively large effective masses in the *xz* plane (see Table S1). Therefore, we argue that dimensionless Fröhlich polaron constants for electron states on the band edges are so large that one has to go beyond the assumption of many-body perturbation theory to deal with the electron–phonon interaction and consequently ex–ph interaction.

13-4 *GW* calculation for bulk SiP_2 lattice with optical phonon modes

To demonstrate the strength of the coupling of electron–phonon matrix elements, we performed *GW* calculations for bulk SiP_2 with optical phonon modes. This is because the size of the splitting (due to the frozen atomic displacements) is directly proportional to the strength of electron–phonon coupling. Specifically, the slope in the change of the energy with respect to the atomic displacements is given by a specific single electron–phonon matrix element, which is obtained from frozen-phonon calculations in a supercell[62, 63]. Therefore, for a given set of atomic displacements, the larger the splitting of the energy level is, the larger the electron–phonon matrix element is, and the shifts of the energy bands induced by the phonon displacements could be regarded as the reduced electron–phonon matrix element (REPME)[63]. This method has been widely used to evaluate the electron–phonon interaction strength in the field of *ab initio* calculations.

In the paper from Li et al.[62], the authors have shown that the REPME calculated from *GW* calculation within the frozen phonon approach has the same level of accuracy as

perturbation theory based on *GW* calculations (*GW*PT). Therefore, we use the same method to estimate REPME from *GW* calculations within the frozen phonon approach.

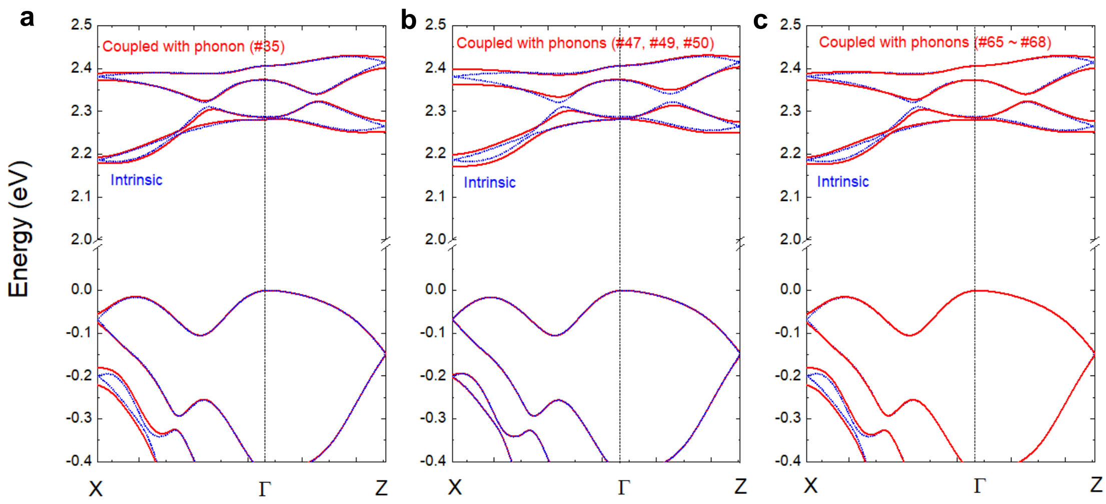

Figure S26 Electronic band structures from *GW* calculations with and without atomic displacements along the direction of optical phonon modes. The blue dotted lines represent bands without atomic displacements. The red solid lines are bands with positive and negative atomic displacements for **a**, optical phonon mode #35, **b**, the sum of phonon modes #47, #49 and #50, and **c**, the sum of phonon modes from #65 to #68. We set the energy of the valence band edge at the Γ point to zero. The displacement of optical phonon modes $\pm\boldsymbol{\chi}_{\nu}(\mathbf{q}=0)$ is obtained by using the same methods discussed in Sec. 13-2.

Figure S26 shows the electronic band structures obtained from *GW* calculations with and without atomic displacements $\pm\boldsymbol{\chi}_{\nu}(\mathbf{q}=0)$ along the directions of selected optical phonon modes. The phonon calculations are performed by using the exchange-correlation functional with vdWs correction to obtain atomic displacement $\pm\boldsymbol{\chi}_{\nu}(\mathbf{q}=0)$. Then, we perform *GW* calculations with lattice structures containing displacements of $\pm\boldsymbol{\chi}_{\nu}(\mathbf{q}=0)$ for the ν-th optical phonons. These calculated results show that the electronic states around the Fermi level can be modified by coupling to optical phonon modes, such as the degeneracy at the high symmetric points being lifted and the band

gap changing correspondingly. We find that E_g^{X} induced by the same optical phonon modes obtained from *GW* calculations is larger than that from conventional DFT calculations (see Fig. 2e in the Main Text and Sec. 13-2), which indicates that the many-body interaction is likely to enhance the electron–phonon coupling in bulk SiP_2 and results in a relatively larger ex–ph interaction.

References

1. Bader, R. F. W. *Atoms in molecules: a quantum theory*. (Oxford: Clarendon press, 1990).
2. Lefebvre, J., Fraser, J. M., Finnie, P. & Homma, Y. Photoluminescence from an individual single-walled carbon nanotube. *Phys. Rev. B* **69**, 075403 (2004).
3. Wang, F., Dukovic, G., Brus, L. E. & Heinz, T. F. The optical resonances in carbon nanotubes arise from excitons. *Science* **308**, 838–841 (2005).
4. Wang, X. et al. Highly anisotropic and robust excitons in monolayer black phosphorus. *Nat. Nanotechnol.* **10**, 517–521 (2015).
5. Aslan, O. B., Chenet, D. A., van der Zande, A. M., Hone, J. C. & Heinz, T. F. Linearly polarized excitons in single- and few-layer ReS_2 Crystals. *ACS Photonics* **3**, 96–101 (2016).
6. Arora, A. et al. Highly anisotropic in-plane excitons in atomically thin and bulklike 1T′-$ReSe_2$. *Nano Lett.* **17**, 3202–3207 (2017).
7. Marulanda, J. M. & Srivastava, A. Carrier density and effective mass calculations in carbon nanotubes. *Phys. stat. sol. (b)* **245**, 2558–2562 (2008).
8. Qiao, J., Kong, X., Hu, Z.-X., Yang, F. & Ji, W. High-mobility transport anisotropy and linear dichroism in few-layer black phosphorus. *Nat. Commun.* **5**, 4475 (2014).
9. Zhong, H.-X., Gao, S., Shi, J.-J. & Yang, L. Quasiparticle band gaps, excitonic effects, and anisotropic optical properties of the monolayer distorted 1T diamond-chain structures ReS_2 and $ReSe_2$. *Phys. Rev. B* **92**, 115438 (2015).

10. Kim, B. S., Kyung, W. S., Denlinger, J. D., Kim, C. & Park, S. R. Strong one-dimensional characteristics of hole-carriers in ReS_2 and $ReSe_2$. *Sci. Rep.* **9**, 2730 (2019).

11. Lee, Y. et al. Atomic-scale imaging of few-layer black phosphorus and its reconstructed edge. *J. Phys. D: Appl. Phys.* **50**, 084003 (2017).
12. Han, B. Crystal growth and characterization of a noval IV-V group two-dimensional semiconductor of SiP_2, Shandong University (2019).
13. Anni, M. et al. Defect-assisted photoluminescence intensity enhancement in poly (*p*-phenylene vinylene) films probed by time-resolved photoluminescence. *Phys. Rev. B* **68**, 035215 (2003).
14. Tongay, S. et al. Defects activated photoluminescence in two-dimensional semiconductors: interplay between bound, charged and free excitons. *Sci. Rep.* **3**, 2657 (2013).
15. Steinhoff, A. et al. Efficient excitonic photoluminescence in direct and indirect band gap monolayer MoS_2. *Nano Lett.* **15**, 6841–6847 (2015).
16. Dey, P. et al. Optical coherence in atomic-monolayer transition-metal dichalcogenides limited by electron-phonon interactions. *Phys. Rev. Lett.* **116**, 127402 (2016).
17. Selig, M. et al. Excitonic linewidth and coherence lifetime in monolayer transition metal dichalcogenides. *Nat. Commun.* **7**, 13279 (2016).
18. Niehues, I. et al. Strain control of exciton–phonon coupling in atomically thin semiconductors. *Nano Lett.* **18**, 1751–1757 (2018).
19. Shree, S. et al. Observation of exciton-phonon coupling in $MoSe_2$ monolayers. *Phys. Rev. B* **98**, 035302 (2018).
20. Mishra, H., Bose, A., Dhar, A. & Bhattacharya, S. Exciton-phonon coupling and band-gap renormalization in monolayer WSe_2. *Phys. Rev. B* **98**, 045143 (2018).
21. Paleari, F., P. C. Miranda, H., Molina-Sánchez, A. & Wirtz, L. Exciton-Phonon Coupling in the Ultraviolet Absorption and Emission Spectra of Bulk Hexagonal Boron Nitride. *Phys. Rev. Lett.* **122**, 187401 (2019).

22. Cudazzo, P. First-principles description of the exciton-phonon interaction: A cumulant approach. *Phys. Rev. B* **102**, 045136 (2020).
23. Mak, K. F. et al. Tightly bound trions in monolayer MoS_2. *Nat. Mater.* **12**, 207–211 (2013).
24. Liu, X. et al. Strong light–matter coupling in two-dimensional atomic crystals. *Nat. Photon.* **9**, 30–34 (2015).
25. Hsu, C. et al. Thickness-dependent refractive index of 1L, 2L, and 3L MoS_2, $MoSe_2$, WS_2, and WSe_2. *Adv. Opt. Mater.* **7**, 1900239 (2019).
26. Ho, C. H., Huang, Y. S., Tiong, K. K. & Liao, P. C. Absorption-edge anisotropy in ReS_2 and $ReSe_2$ layered semiconductors. *Phys. Rev. B* **58**, 16130–16135 (1998).
27. Ross, J. S. et al. Electrical control of neutral and charged excitons in a monolayer semiconductor. *Nat. Commun.* **4**, 1474 (2013).
28. Li, Y. et al. Measurement of the optical dielectric function of monolayer transition-metal dichalcogenides: MoS_2, $MoSe_2$, WS_2, and WSe_2. *Phys. Rev. B* **90**, 205422 (2014).
29. Kuzmenko, A. B. Kramers–Kronig constrained variational analysis of optical spectra. *Rev. Sci. Instrum.* **76**, 083108 (2005).
30. Hecht, E. Optics 4th edition. *Optics 4th edition by Eugene Hecht Reading, MA: Addison-Wesley Publishing Company, 2001* **-1** (2001).
31. Chernikov, A. et al. Exciton binding energy and nonhydrogenic Rydberg series in monolayer WS_2. *Phys. Rev. Lett.* **113**, 076802 (2014).
32. Jusserand, B., Poddubny, A. N., Poshakinskiy, A. V., Fainstein, A. & Lemaitre, A. Polariton resonances for ultrastrong coupling cavity optomechanics in GaAs/AlAs multiple quantum wells. *Phys. Rev. Lett.* **115**, 267402 (2015).
33. Kira, M. & Koch, S. W. *Semiconductor Quantum Optics*. (Cambridge University Press, 2011).
34. Kira, M., Jahnke, F. & Koch, S. W. Microscopic theory of excitonic signatures in semiconductor photoluminescence. *Phys. Rev. Lett.* **81**, 3263–3266 (1998).
35. Viña, L., Logothetidis, S. & Cardona, M. Temperature dependence of the dielectric function of germanium. *Phys. Rev. B* **30**, 1979–1991 (1984).

36. Giustino, F. Electron-phonon interactions from first principles. *Rev. Mod. Phys.* **89**, 015003 (2017).

37. Sarswat, P. K. & Free, M. L. A study of energy band gap versus temperature for Cu_2ZnSnS_4 thin films. *Physica B: Condens. Matter* **407**, 108–111 (2012).

38. Shreve, A. P. *et al.* Determination of exciton-phonon coupling elements in single-walled carbon nanotubes by raman overtone analysis. *Phys. Rev. Lett.* **98**, 037405 (2007).

39. Torrens, O. N., Zheng, M. & Kikkawa, J. M. Energy of K-momentum dark excitons in carbon nanotubes by optical spectroscopy. *Phys. Rev. Lett.* **101**, 157401 (2008).

40. Lüer, L. *et al.* Coherent phonon dynamics in semiconducting carbon nanotubes: A quantitative study of electron-phonon coupling. *Phys. Rev. Lett.* **102**, 127401 (2009).

41. Koo, S. *et al.* Extraordinary photostability and Davydov splitting in BN-sandwiched single-layer Tetracene molecular crystals. *Nano Lett.* **21**, 6600–6608 (2021).

42. Ellis, S. R. *et al.* Resonance raman characterization of Tetracene monomer and nanocrystals: Excited state lattice dstortions with implications for efficient singlet fission. *J. Phys. Chem. A* **123**, 3863–3875 (2019).

43. Smith, M. *et al.* Exciton-phonon interaction in InGaN/GaN and GaN/AlGaN multiple quantum wells. *Appl. Phys. Lett.* **70**, 2882–2884 (1997).

44. Smith, M. *et al.* Optical transitions in GaN/$Al_xGa_{1-x}N$ multiple quantum wells grown by molecular beam epitaxy. *Appl. Phys. Lett.* **69**, 2453–2455 (1996).

45. Umlauff, M. *et al.* Direct observation of free-exciton thermalization in quantum-well structures. *Phys. Rev. B* **57**, 1390–1393 (1998).

46. Zhao, H., Moehl, S. & Kalt, H. Coherence length of excitons in a semiconductor quantum well. *Phys. Rev. Lett.* **89**, 097401 (2002).

47. Xu, S. J. *et al.* Spectral features of LO phonon sidebands in luminescence of free excitons in GaN. *J. Chem. Phys.* **122**, 244712 (2005).

48. Yeo, Y. C., Chong, T. C. & Li, M. F. Electronic band structures and effective-mass parameters of wurtzite GaN and InN. *J. Appl. Phys.* **83**, 1429–1436 (1998).

49. Feldtmann, T., Kira, M. & Koch, S. W. Phonon sidebands in semiconductor luminescence. *Phys. Stat. Sol. (b)* **246**, 332–336 (2009).

50. Jang, S.-H. & Chichibu, S. F. Structural, elastic, and polarization parameters and band structures of wurtzite ZnO and MgO. *J. Appl. Phys.* **112**, 073503 (2012).

51. Cannuccia, E., Monserrat, B. & Attaccalite, C. Theory of phonon-assisted luminescence in solids: Application to hexagonal boron nitride. *Phys. Rev. B* **99**, 081109 (2019).

52. Ward, L., Agrawal, A., Choudhary, A. & Wolverton, C. A general-purpose machine learning framework for predicting properties of inorganic materials. *npj Comput. Mater.* **2**, 16028 (2016).

53. Wang, Y. et al. A mixed-space approach to first-principles calculations of phonon frequencies for polar materials. *J. Phys.: Condens. Matter* **22**, 202201 (2010).

54. Zhong, W., King-Smith, R. D. & Vanderbilt, D. Giant LO-TO splittings in perovskite ferroelectrics. *Phys. Rev. Lett.* **72**, 3618–3621 (1994).

55. Lasota, C., Wang, C.-Z., Yu, R. & Krakauer, H. Ab initio linear response study of $SrTiO_3$. *Ferroelectrics* **194**, 109–118 (1997).

56. Ghosez, P., Michenaud, J. P. & Gonze, X. Dynamical atomic charges: The case of ABO_3 compounds. *Phys. Rev. B* **58**, 6224–6240 (1998).

57. Shah, S. H., Bristowe, P. D., Kolpak, A. M. & Rappe, A. M. First principles study of three-component $SrTiO_3$/$BaTiO_3$/$PbTiO_3$ ferroelectric superlattices. *J. Mater. Sci.* **43**, 3750–3760 (2008).

58. Capaz, R. B., Spataru, C. D., Tangney, P., Cohen, M. L. & Louie, S. G. Temperature dependence of the band gap of semiconducting carbon nanotubes. *Phys. Rev. Lett.* **94**, 036801 (2005).

59. Poncé, S. et al. Temperature dependence of electronic eigenenergies in the adiabatic harmonic approximation. *Phys. Rev. B* **90**, 214304 (2014).

60. Antonius, G. & Louie, S. G. Theory of the exciton-phonon coupling. Preprint at https://arxiv.org/abs/1705.04245 (2017).

61. Mahan, G. D. *Many-particle physics*. (Springer Science & Business Media, 2013).

62. Li, Z., Antonius, G., Wu, M., da Jornada, F. H. & Louie, S. G. Electron-phonon coupling from ab initio linear-response theory within the GW method: correlation-enhanced interactions and superconductivity in $Ba_{1-x}K_xBiO_3$. *Phys. Rev. Lett.* **122**, 186402 (2019).

63. Yin, Z. P., Kutepov, A. & Kotliar, G. Correlation-enhanced electron-phonon coupling: applications of GW and screened hybrid functional to bismuthates, chloronitrides, and other high-T_c superconductors. *Phys. Rev. X* **3**, 021011 (2013).
